# Complementary Analysis and Implementation Plan for Conservation of Crop Wild Relatives in Finland

**DOI:** 10.3390/plants12183313

**Published:** 2023-09-19

**Authors:** Heli Fitzgerald, Elina Kiviharju, Anna Palmé, Marko Hyvärinen

**Affiliations:** 1 Botany Unit, Finnish Museum of Natural History, LUOMUS, University of Helsinki, P.O. Box 7, 00014 Helsinki, Finland; marko.hyvarinen@helsinki.fi; 2 Natural Resources Institute Finland, Luke, 31600 Jokioinen, Finland; elina.kiviharju@luke.fi; 3 Nordic Genetic Resource Center, NordGen, 234 56 Alnarp, Sweden; anna.palme@nordgen.org

**Keywords:** crop wild relatives, plant genetic resources, in situ conservation, ex situ conservation

## Abstract

Crop wild relatives (CWR) are valuable wild plant species that can be used as genetic resources providing adaptive traits to crop plants and therefore they play an important role in future food security. This paper describes in situ and ex situ conservation planning of CWR species in Finland and includes the following parts: (a) drafting of the national CWR priority list, (b) undertaking the in situ conservation gap analysis and (c) identifying ex situ conservation gaps and multi-species collecting sites for the CWR in Finland. As a result of the study, essential information was acquired, which will enhance future planning of active science-based practical conservation of CWR in Finland. Based on the new data and earlier work, a number of conservation recommendations are presented. This national work has been carried out in connection with the larger Nordic regional CWR co-operation.

## 1. Introduction

Data from The State of Food and Agriculture—Climate Change, Agriculture and Food Security [1] show that crop plants are affected by new extreme conditions induced by climate change and harvests are expected to be reduced. This, along with a rapidly growing world population threatens global food security, thus there is a need for a prompt solution. Crop wild relatives (CWR) have developed evolutionary adaptations to the environmental conditions in their wild habitats and therefore frequently harbour useful traits such as the resistance to pests and diseases, and the ability to withstand waterlogging, drought or extreme temperatures. CWR can therefore be used in plant breeding to add much-needed traits such as these to crops [2,3]. However, the CWR species that can play a part in providing solutions for food security are themselves threatened or under pressure due to unsustainable land use, urban development, effects of climate change, habitat loss and fragmentation [4,5]. Their genetic and phenotypic diversity is poorly known and as the conservation of CWR falls between the agricultural and environmental sectors, they are not specifically noted in species conservation programmes.

The importance of conservation of wild relatives is recognized in international treaties such as The Strategic Plan for Biodiversity 2011–2020 [6,7], the first draft of the post-2020 global biodiversity framework [8], The International Treaty on Plant Genetic Resources for Food and Agriculture [9], The Global Plan of Action for Plant Genetic Resources for Food and Agriculture [10] and The Sustainable Development Goals [11] but so far there have been limited practical conservation actions. In Finland, the National Genetic Resources Programme [12] recognizes CWR and underlines that further actions are needed to ensure their conservation.

In this paper, we describe the results from a project titled ‘Developing conservation strategy for Finnish wild relatives and finding practical conservation options’ dedicated to finding conservation solutions for Finnish wild plant species of importance to regional and global food security by developing different ways of in situ (in wild habitats) and ex situ (in genebanks) conservation and finding options for their practical implementation. The goal was to find cost-efficient CWR conservation approaches by identifying the most diverse and complementary in situ conservation areas and ex situ collecting sites through gap analysis. Complementary conservation and collecting sites enable the conservation of maximum diversity in a minimum number of sites. Representativeness analysis compared the existing genebank collections with the diversity in the wild and complementarity analysis identified sites where, by collecting, the ex situ gaps could be filled. The creation of the second and updated version of the CWR priority list formed the base of the in situ and ex situ conservation analyses. The new priority list will enable better harmonization of conservation actions between Finland and the Nordic region. This research provides valuable information in Finland for implementing conservation of CWR diversity.

## 2. Materials and Methods

### 2.1. National CWR Checklist and Prioritization

The Nordic CWR checklist [13] and the Nordic priority list [14] were used as the basis of the new Finnish priority list. The Nordic checklist was prioritized according to the utilization potential and socio-economic use categories. This was achieved by first selecting the wild relatives to food and forage crops; second by considering the socio-economic value of the related crop by using the FAO production values of crop genera; and third by applying the gene pool [15] and taxon group [16] concepts to the taxa. Those taxa belonging to the primary and secondary gene pool and taxon group of the crop were prioritized along with the ones in the tertiary gene pool with proven use potential in plant breeding.

**Figure 1:**
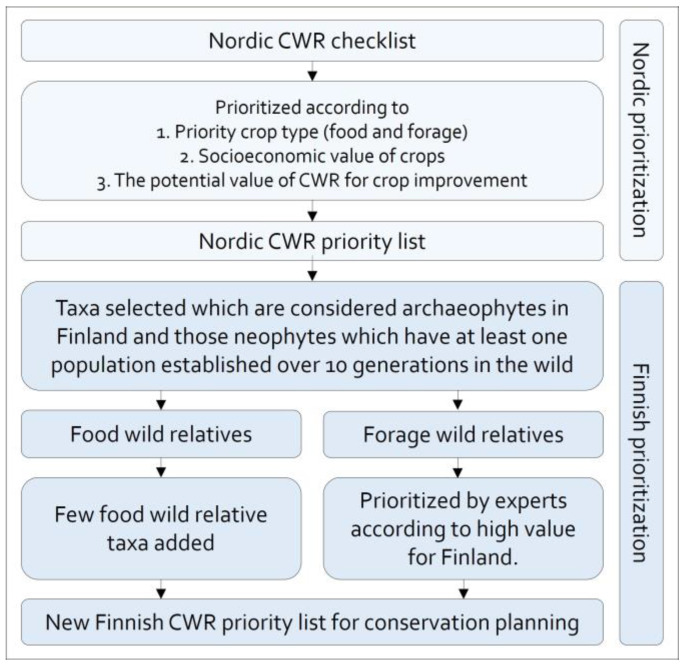
Summary of second Finnish CWR priority list development.

The original Finnish priority list [17] included also threat status as a prioritization criterion. That was not included this time as much more data on utilization potential were available and because many threatened species already have conservation actions in place. Those taxa related to food crops from the Nordic priority list that are present in Finland as archaeophytes or established neophytes were included in the new Finnish priority list. The taxa in the Nordic priority list which do not occur in Finland, only occur in Finland as escapees from cultivation or have been established in the wild less than 10 generations were not included. The forage wild relatives were also considered a priority in Finland but were re-prioritized by national experts in accordance with their importance to the national forage cultivation and breeding at present and potentially in the future. In addition to the changes regarding forage CWR priorities, a few food wild relative species were added to the new Finnish list which were on the original Finnish list but not on the Nordic priority list as they were considered important by national experts. The process is summarized in Figure 1. After prioritization, the national threat status of the priority taxa was checked as the threat status may affect conservation actions.

### 2.2. In Situ Conservation Analysis

The aim of the in situ analysis was to find diverse and complementary CWR sites to be established as genetic reserves within existing protected areas (PA) in Finland. Two separate analyses were carried out, one for mainland Finland and another which included both the mainland and the autonomic archipelago region, Åland Islands. The analyses of two networks of genetic reserve sites offer alternative options during future implementation because Åland Islands and mainland Finland have separate environmental administrations. The in situ conservation analysis was based on the principle of complementarity when selecting conservation sites [18,19] and on using ecogeographic diversity as a proxy of genetic diversity areas [20]. The complementarity of the in situ sites means that the CWR diversity in the network complements each other. We aimed to identify those priority CWR populations in a network of sites, which represents the maximum species ecogeographic diversity in a minimum number of sites. Capfitogen tools “ELCmapas” and “Complementa” [21] were used in the analysis. The Finnish species distribution data [22] were filtered for the CWR taxa and the most recent observations made after 1990 from each observation point with a minimum of 1 km^2^ accuracy. Duplicate records were removed, and outlier locations were moved when necessary. The outlier locations were coastline observations originally recorded in a grid system and for which the centroids of the grid were calculated. With coastal points, some grid centroids were outside the land boundary. These were assigned the closest coordinate point on the boundary if they were less than 1 km from the boundary line. After the filtering process, the species observations of the 56 priority species were used in the in situ conservation analysis.

A generalist ecogeographic land characterization (ELC) map for the Finnish CWR priority species was made with the ELCmapas tool of Capfitogen tools based on the information of priority CWR. The same non-correlated variables were used as in the Nordic in situ conservation analysis [23] and were checked by national experts to suit Finland. These included two bioclimatic variables: average annual rainfall and average annual temperature [24,25,26]; four geophysical variables: elevation, slope, and north and east aspects [27,28] and three edaphic variables: soil depth, topsoil organic carbon content and pH in soil water solution of surface soils [29]. The parameters used in the Capfitogen ELCmapas tool were 30 arc-s (~1 km^2^) spatial resolution, Calinski method, latitude, 40 iterations and three clusters allowed by each component.

The PA complementary analyses were used to identify a minimum number of sites within existing PAs complementary to each other with a maximum number of CWR species, taking into consideration the ecogeographic diversity identified in the ELC map categories (i.e., species/ELC category combinations). The analyses were conducted with the Complementa tool of the Capfitogen tools. For the mainland Finland analysis, protected area shapefiles from Parks and Wildlife Finland were used. These included the existing national PA designations and those areas planned to be protected within the near future. For the analysis including Åland Islands, the World Database of Protected Areas [30] was used on the resolution of 30 arc-s (~1 km^2^). This dataset also included regional and international PA designations, such as UNESCO Biosphere Reserves, HELCOM and Ramsar sites.

### 2.3. Ex Situ Conservation Analysis

The ex situ conservation gap analysis aimed to find out to what extent the Finnish priority CWR are conserved ex situ and how representative the conserved ex situ seed collections are compared to the ecogeographic diversity within wild populations. Both mainland Finland and Åland Islands were considered as one area in the ex situ gap analysis. Contrary to the in situ analysis, there was no need to make separate plans for ex situ collecting plan because there are existing seedbanks/genebanks already established for ex situ storage and seed collecting can be organized in both areas based on a joint plan.

Ex situ gap analysis included on one hand, those species which already have some conserved ex situ accessions, and on the other hand, those which have not (Table 1). Capfitogen tools ELCmapas, SelecVar, Representa and Complementa [21] (Parra-Quijano, 2016) were used to determine multi-species complementary collecting sites. The ex situ conservation analysis was based on ecogeographic diversity as a proxy for genetic diversity when determining ecogeographic gaps in genebank collections, as described in [31] where prioritized collection sites were found using ELC maps and gap analysis. These gaps are planned to be filled by optimized collecting design [32], improving the ecogeographic representativeness of ex situ collections.

The Finnish wild plant species are conserved ex situ in two main locations: Finnish Threatened Species Seedbank in the Finnish Museum of Natural History, Helsinki (safety duplicated in the Millenium seedbank) and the Nordic genebank, NordGen in Alnarp, Sweden (safety duplicated in NordGen’s backup seedbank in Denmark and Svalbard Seed Vault). The ex situ representativeness gap analysis compared the seedbank collection data [33,34] with the priority CWR list to find out how many populations had been collected and from where. Only the accessions in long-term conservation which had collection site latitude and longitude data were included in the analysis. Those 18 priority species, with seed accessions in the genebanks, were selected for the representativeness analysis. The remaining 37 priority CWR species (Table 1) were included in a complementary collecting site planning. The representativeness analysis was carried out in three stages: 1. selection of variables for the species-specific ELC maps with Capfitogen SelecVar tool, 2. generation of species-specific ELC maps with Capfitogen ELCmapas tool, and 3. identification of ecogeographical gaps with Capfitogen Representa tool. After this, the complementarity analysis with the Capfitogen Complementa tool was used to identify the complementary ecogeographic gaps population sites for future collections of both the species with ex situ accessions and the species with no ex situ accessions.

The variable selection for species-specific ELC maps was conducted using the Capfitogen SelecVar tool. The most relevant non-correlated variables were selected for each species from bioclimatic, edaphic, and geophysic variables by random forest classification (RFC) and Pearson correlation coefficients using a similar methodology as the multi-species collecting strategy in [32]. Variable importance for each species was calculated with RFC, this was conducted separately for bioclimatic and edaphic variables, which ranked them in ‘mean decrease accuracy’ value order. The geophysical variables were not calculated by SelecVar since only four—elevation, aspect, eastness and northness—were included in the ex situ conservation analyses. The Pearson correlation coefficients were similarly calculated for the bioclimatic and edaphic variables. Pairs of the top four variables from RFC results were compared and selected if their correlation coefficients were <|0.50| (indicating low or no correlation) and the *p*-value > 0.05. If the correlation coefficients were >|0.50| (indicating high correlation) and *p*-value < 0.05, the lower-ranked variable in RFC was removed.

Species-specific ELC maps were created with the Capfitogen ELCmapas tool for the taxa with ex situ accessions. The four most important and non-correlated bioclimatic and edaphic variables indicated by SelecVar analysis were used, as well as the four selected geophysical variables. The ELC maps were made in 5 arc-min cell size with the following parameters: maximum of three clusters per ecogeographical component, Calinski-Harabasz procedure and 40 iterations. A generalist ELC map with the same variables and parameters as the in situ gap analysis was used in the ex situ analysis for those 37 CWR species which had no accessions with adequate documentation and conservation status in genebanks and were therefore not included in the representativeness analysis. A generalist ELC map was developed and used in complementarity in situ and ex situ analyses. Generalist ELC maps have been used in several other CWR complementarity analyses, such as in [32,35,36,37,38,39], and are especially useful when occurrence data for individual species are inadequate with a low number of occurrence records, which is the case for several of the species analysed here (less than 30 occurrence records).

Ecogeographic representativeness analysis [40] was used to identify ecogeographic gaps of those CWR species populations which have accessions in ex situ seed conservation to see how representative the ex situ collections are. Ecogeographic gaps were identified with the Capfitogen Representa tool using species-specific ELC maps and the in situ and ex situ species occurrence points. Ecogeographic gaps were identified as those occurrence points within an ecogeographic category that were not represented in genebank collections. A species’ ecogeographic diversity is considered adequately conserved ex situ when the collection has at least one accession from each of the species ELC categories. We considered occurrences with ecogeographic gap high-priority levels 1–4, from Capfitogen Representa analysis, as a priority for future collecting. These classes were the result of Representa analysis to find the priority level of each ecogeographic category for future collection starting from class 1 as the highest priority.

The occurrences classified as high-priority ecogeographic gaps from the 18 priority species and the occurrences for those priority species not in ex situ conservation were observed as ecogeographic gaps. The ecogeographic gap occurrences were included in the ex situ complementarity analysis to find complementary sites of priority gap populations for future collecting. The complementary analysis was conducted with the Capfitogen Complementa tool similar to the in situ conservation gap analysis but this time identifying multi-species collecting sites from the whole land area of Finland, and not only within conservation areas as in the in situ analysis, on a 10 × 10 km grid system.

## 3. Results

### 3.1. CWR Prioritization

The new Finnish CWR priority list includes 88 taxa, representing 56 species from 31 genera. The priority taxa and information on their inclusion in in situ and ex situ analyses can be found in Table 1 and the priority list with additional data on the name of the crop the CWR is related to gene pools, taxon groups, nativity, and vernacular names can be found in [41].

**Figure 2:**
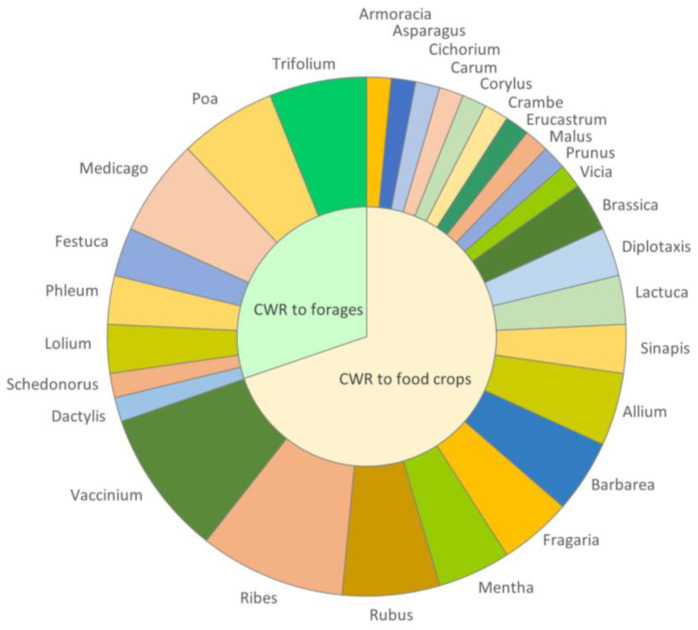
Finnish priority CWR genera in the food and forage groups.

The list includes relatives to vegetable, fruit, berry, nut, spice, and forage grass and legume crop groups. The wild relatives of vegetables are in the primary or secondary gene pool of, for example: cabbage, broccoli, cauliflower, chives, asparagus, turnip, endive and broad bean. Some of the priority CWR are closely related to fruits and/or berries such as cultivated apples, plums, currants, gooseberries, raspberries and blueberries. The list also includes species related to nuts and spices, such as hazelnut, caraway, chives, mustard and mints. Most important forage legumes and grasses for breeding, such as fescues, ryegrasses, bluegrasses, clovers, medicks and timothies are included. All the genera in the priority list grouped into food and forage categories are shown in Figure 2.

Even though threat status was not one of the priority criteria, a few prioritized taxa are listed as Vulnerable in the Finnish red list [42]: *Fragaria viridis* Weston., *Malus sylvestris* Mill., and *Vicia lathyroides* L. Some are listed as Near Threatened: *Allium schoenoprasum* subsp. *sibiricum* (L.) Hartm., *Phleum nodosum* L. and *Mentha aquatica* subsp. *litoralis* Hartm.

### 3.2. ELC Maps and Variable Selection

**Figure 3:**
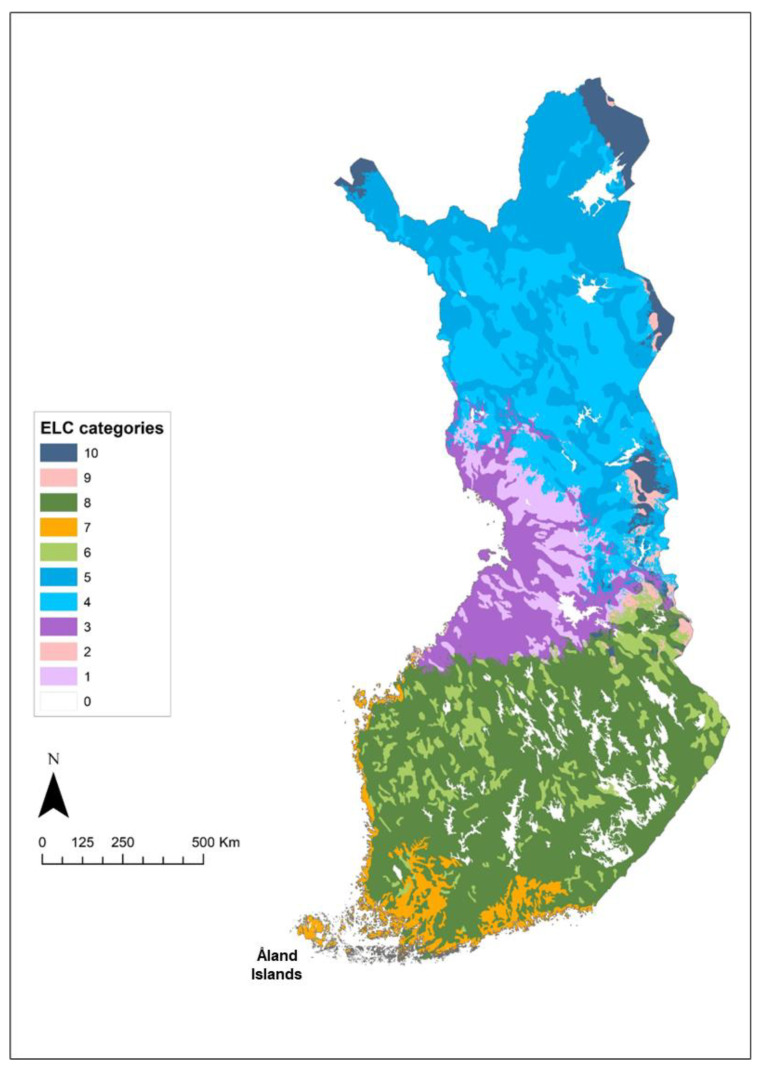
Generalist ecogeographic land characterization (ELC) map created for the priority CWR. ELC category 0 refers to water bodies.

The general ecogeographic land characterization (ELC) map was developed for the 56 Finnish CWR priority species (Figure 3). All conservation planning was conducted on a species rather than taxon level because in that way more comprehensive observation data could be used. The ELC categories were used in the in situ complementary analysis to identify potential genetic reserve sites within existing PAs to maximise the diversity conserved in protected areas, and in the ex situ complementary analysis to find multi-species collecting sites for filling the gaps and to maximise the diversity in ex situ seed collections. See Supplementary Material Table S1 for information on the ELC categories.

In addition to the generalist ELC map, species-specific maps were developed for the species in ex situ representativeness analysis. The variables used in creating species-specific ELC maps and categories are shown in Appendix Table A1 and the average values of variables in Table S1. The most important bioclimatic variables for the 18 taxa were annual average temperature, rainfall during the wettest quarter and temperature seasonality (in 10, 8 and 8 taxa, respectively). The silt content in surface soil, percentage of exchangeable sodium in the topsoil and clay cation exchange capacity in surface soil were the most important edaphic variables (in 10, 9 and 7 taxa, respectively).

### 3.3. In Situ Conservation Analysis

Two in situ analyses were made: one for the PAs in mainland Finland and another including the PAs in mainland Finland and the Åland Islands. A few of the priority CWR have their main Finnish distribution in the Åland Islands: *Mentha aquatica* L., *Malus sylvestris* (L.) Mill., *Vicia lathyroides* L. and *Rubus caesius* L. Therefore, these priority species were only included in the Åland Islands analysis. In total, after the filtering process described in the materials and methods, 280,372 species observations of the 56 priority species were used in the in situ conservation analysis.

**Figure 4:**
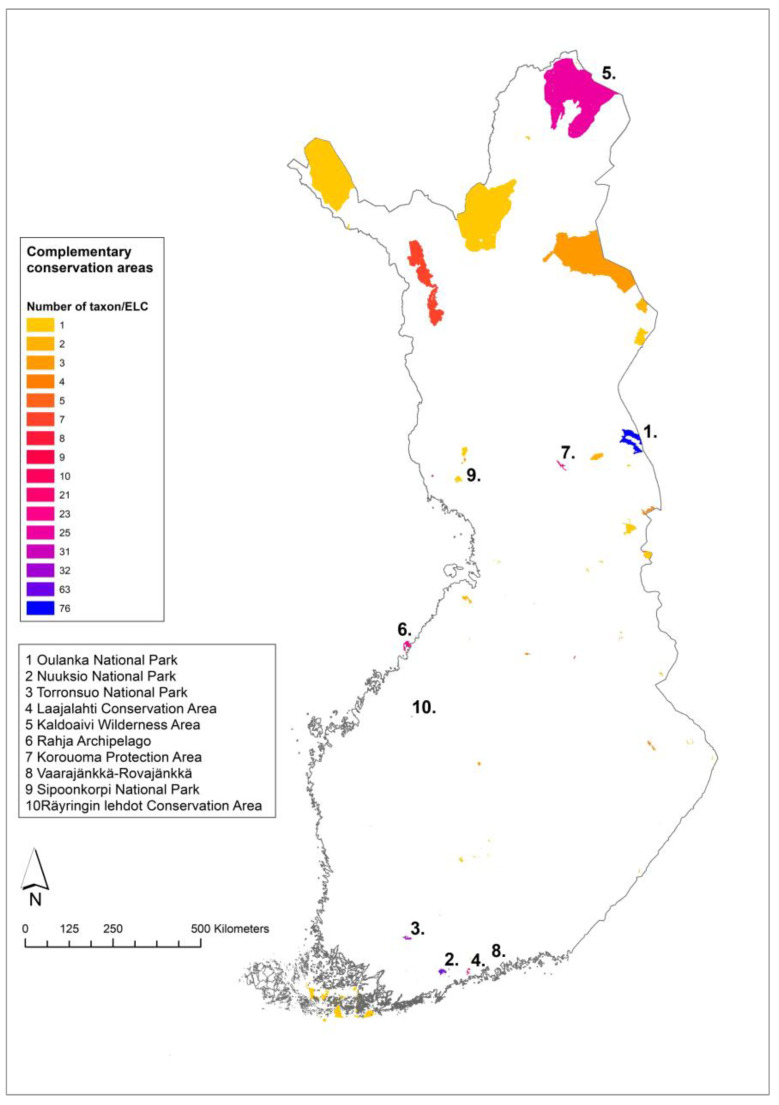
Complementary network of in situ sites identified in gap analysis for mainland Finland with the top 10 sites numbered.

The mainland Finland complementarity analysis resulted in 59 complementary sites within protected areas (Table A2). Out of 56 CWR, 9 species had no observations within protected areas: *Asparagus officinalis* L., *Chenopodium ficifolium* Sm., *Cichorium intybus* L., *Diplotaxis muralis* (L.) DC., *Diplotaxis tenuifolia* (L.) DC., *Erucastrum gallicum* (Willd.) O. E. Schulz, *Lactuca tatarica* (L.) C. A. Mey., *Medicago lupulina* L. and *Sinapis arvensis* L. The rest of the target species had altogether 7232 observations within PAs. The top 10 PAs would, if conserved, cover 72% of the species/ELC category combinations (Figure 4). The species and ELC categories of the sites are listed in Table S2.

**Figure 5:**
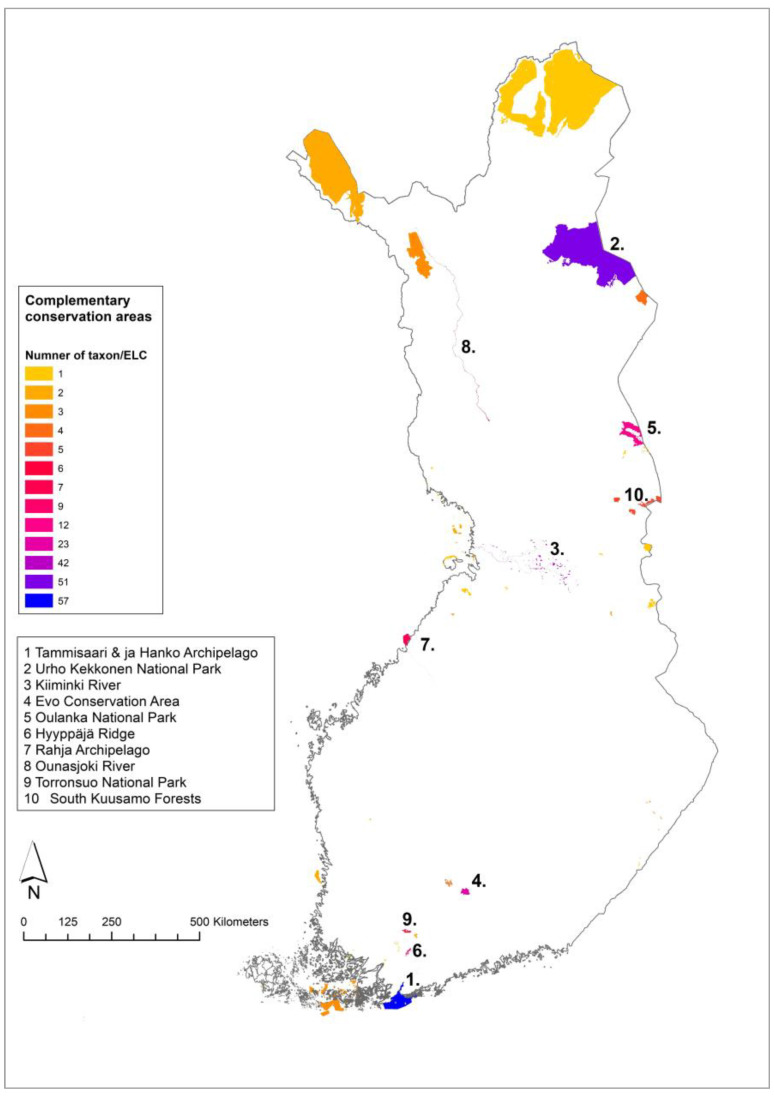
Complementary network of in situ sites identified in gap analysis for Åland Islands and mainland Finland with the top 10 sites numbered.

The mainland Finland and Åland Islands complementary analysis resulted in 49 complementary sites within PAs (Table A3) which would conserve the majority of the Finnish priority CWR in all the ELC zones where they occur, except for the five species having no observations in the PAs: *Chenopodium ficifolium* Sm., *Cichorium intybus* L., *Diplotaxis muralis* (L.) DC., *Diplotaxis tenuifolia* (L.) DC. and *Erucastrum gallicum* (Willd.) O. E. Schulz. The 49 PAs contained 7004 CWR observations of the priority CWR. The top 10 sites, if established as genetic reserves, would conserve 76% of the Finnish priority CWR-ELC combinations (Figure 5). The species/ELC category combinations of the sites are listed in Table S3.

The first and second sites from the mainland Finland analysis were Oulanka National Park with 73 species/ELC combinations and Nuuksio National Park with 62 combinations (Table S2). From the Åland–mainland Finland analysis, Tammisaari and Hanko Archipelago area was first with 57 Finnish complementary species/ELC combinations and Urho Kekkonen National Park second with 51 combinations (Table S3).

### 3.4. Ex Situ Complementary Analysis and Identification of Multi-Species Collecting Sites

**Figure 6:**
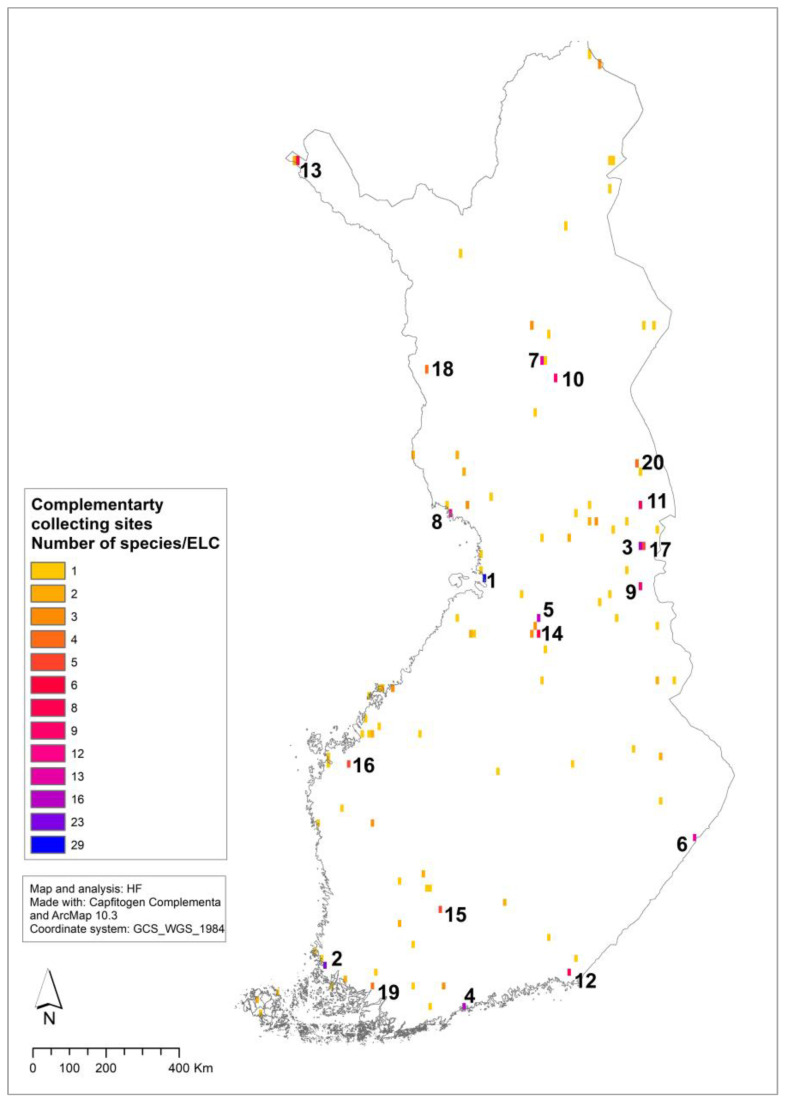
Multi-species seed collecting sites to fill ex situ conservation gaps of the priority CWR species. The top 10 sites numbered on the map (see Table S1 for information on complementary species occurrence).

The 18 priority species (Table 1) included in the representativeness analysis had 188,752 observation points. Out of these, the Capfitogen Representa tool identified 43,320 priority ecogeographical gap points in ecogeographic gap range 1–4. Additionally, for those species not in ex situ conservation, 27,502 species occurrences were observed as ecogeographic gaps. The ecogeographic gap records were joined with species gap records (observation points of those priority species with no ex situ collections) in the Capfitogen Complementa tool and the ELC category data in both gap data types were used in the complementarity analysis to find multi-species complementary collecting sites. The result of the complementarity analysis was 107 multi-species collecting sites on a 5 arc-min (~10 km^2^) spatial resolution within Finland (Figure 6). The 107 sites included altogether 332 species/ELC combinations from 54 species (Table S4). These 332 species/ELC combinations are target species gap populations, which, if collected, would fill the ex situ gaps of Finnish priority CWR. The top 10 of the 332 multi-species collecting sites cover 45% of the species/ELC gaps.

All the 18 species that have accessions in ex situ genebanks need more collecting to conserve the full ecogeographic diversity. These species need to be collected from an average of 6 sites each. Those with large numbers of existing genebank accessions such as *Phleum pratense* L. and *Trifolium pratense* L. (Table 1) and those with distribution in a limited number of ecogeographic categories, such as *Crambe maritima* L. (Table S4), only need one or two more collections. On the other hand, those species with very few genebank accessions, such as *Vaccinium microcarpum* (Turcz. ex Rupr.) Schmalh., *V*. *myrtillus* L., *V. oxycoccos* L., *V. uliginosum* L. and *V. vitis-idaea* L., need additional collections from the rest of the ecogeographic zones. To ease collecting logistics, the multi-species complementary collecting site analysis enables the collecting of many species from necessary ecogeographic categories from the same sites.

## 4. Discussion

### 4.1. Finnish CWR Priority List

The new priority list includes the 88 most important CWR taxa, 56 species from 31 genera, in Finland, which need national in situ and ex situ conservation planning and implementation. The new Finnish CWR priority list differs from the first list [17] due to the increased data on utilization potential on crop improvement, different prioritization methods, availability of the Nordic checklist [13] and priority list [14], and different criteria used. The new list focuses more on utilization potential and less on threat status. The selection of food and forage categories out of different CWR categories such as food, forage, medicinal, forestry and ornamental, reduced the list significantly compared to the first priority list in which CWR of medicinal plants were also included. Many of the future recommendations in the first Finnish CWR conservation report [17] have been considered and solved in this second iteration of the prioritization and conservation gap analyses. These include (1) a creation of a shorter priority list which considers breeders’ views, and potential future use of wild species; (2) taking into consideration the ecogeographic diversity in the gap analyses as a proxy to genetic diversity to allow for variation between different regions and habitats when planning the CWR conservation; (3) co-operation with nature management authorities which took place alongside these analyses during the project ‘‘Developing conservation strategy for Finnish wild relatives and finding practical conservation options’. The report [17] also suggests that the new iteration should take place in 5 years’ time.

Whereas the old list was limited to indigenous and archaeophyte taxa, the new list includes those neophytes which are estimated to have had populations reproducing in the wild for over 10 generations in Finland [41]. Effort has been made to include the most important CWR for utilization and to aim for easier conservation actions with a smaller size priority list, from 209 taxa [17] to 88 taxa. Some species on the new list are listed as Threatened, Near Threatened or Vulnerable in the Finnish Red List [42] even though threat was not used as a separate criterion. Harmonization with the Nordic priority list by creating a second updated national priority list will make regional conservation efforts easier in the future. Differences compared to the Nordic CWR priority list include only a few newly added species such as wild mints *Mentha aquatica* L., and *Mentha arvensis* L. and *Chenopodium ficifolium* Sm.

### 4.2. In Situ Conservation Analysis

The results of the complementary analysis indicate that conservation efforts should first be focused on the top 10 areas (Figure 3 and Figure 4), which cover 72–76% of the CWR diversity. When comparing the results of the national in situ analysis described here and the Nordic regional analysis [23,43], the two analyses largely identify different top sites within Finland. This is likely due to using separate ELC maps and the nature of ecogeographic complementarity analyses, which first select the site with the highest number of species/ELC combinations and then remove that combination from the next step. Therefore, if a species/ELC combination is already selected in another country, it will not be selected from Finland in the Nordic analysis. For the same reason, the mainland Finland and Åland-mainland analyses results were quite different. For the regional complementary conservation to be successful, all the countries/areas should eventually implement CWR conservation in the sites selected. As the CWR conservation planning and implementation stage varies between the countries and areas, the national analysis was deemed important at this stage. Sweden has similarly carried out a national in situ complementarity analysis [36] after being included in the Nordic regional in situ complementary analysis. In the Swedish analysis, three sites were common in both analyses and all other locations differed. The use of a Nordic-level complementary analysis probably offers more efficient conservation of the relevant diversity, but it is based on the assumption of implementation in all the countries.

In Finland, the regional and national results had some overlapping sites, most importantly the Tammisaari and Hanko Archipelago conservation area, which was the top site in the Åland-mainland analysis and fourth in the regional analysis. This shows that the Tammisaari and Hanko area has significant CWR diversity both nationally and regionally. Oulanka National Park was found among the top 10 sites in both mainland and Åland-mainland analyses. The second complementary sites from the two Finnish analyses were Nuuksio National Park and Urho Kekkonen National Park. These four sites provide a good starting point for future CWR conservation implementation in Finland. The best practices for inventory, monitoring and managing CWR diversity need to be defined and they should be included in the management plans of the PAs. The first step forward is to carry out detailed inventories in the top sites to identify target populations for conservation action.

The nine priority taxa growing only outside PAs also need further investigation. Studies on the habitat preferences of CWR suggest that many taxa are found in disturbed areas such as roadsides and field margins [44], which are not generally part of the prioritized features of PAs. The five taxa that had no observations in PAs either in the mainland or the mainland and Åland datasets (*Chenopodium ficifolium*, *Cichorium intybus*, *Diplotaxis muralis*, *Diplotaxis tenuifolia*, *and Erucastrum gallicum*) all occur in disturbed habitats in the Nordic region [45], suggesting that this could be an important reason for their non-inclusion. The fact that many CWR are associated with disturbed habitats can increase their vulnerability long-term, both because these habitats are not prioritized by nature conservation agencies and because they are highly affected by anthropogenic-induced changes [2]. It is therefore important to identify an alternative approach for the CWR occurring mainly in disturbed habitats. This being said, a great majority of the prioritized CWR have been found within PAs in Finland and efforts targeted at these areas would conserve a large proportion of the CWR diversity.

### 4.3. Ex Situ Conservation Analysis

The ex situ analysis identifies multi-species complementary collection sites to fill the gaps in existing genebank collections. In total, 332 species/ELC combinations from 54 CWR priority species are suggested for targeted collections from 107 collecting sites. The results provide the possibility to plan targeted collecting trips for CWR ex situ gaps and enable the collection of many species from different ecogeographic categories from the same area. The top collecting sites include a larger number of gaps because of the complementary nature of the analysis. By collecting from the top 10 sites, 45% of the ecogeographic gaps would be conserved and the top 20 sites would capture 61% of the gaps.

Further planning is still needed before collection as the analysis does not give information on population size and suitability or the land use of the target collecting sites. Even with multi-species sites, the CWR seeds can still mature at different times, so a few visits to each site might be required depending on the species. In addition, the collection of seeds for ex situ conservation within other projects or as backups of conservation of populations in the in situ conservation sites can fill some of the current gaps by sampling in other locations. Over time it would be beneficial to re-do the gap analysis at intervals, each time including new information and data on newly collected samples.

Ex situ conservation is important both as a backup of conservation in situ and for providing efficient access to genetic resources. The Nordic gene bank is a part of NordGen and offers access to all accessions in their active collection, both to Nordic and international users. The Kalmar declaration [46], which has been signed by the Nordic countries, states that “all accessions of the Nordic Gene bank, except for security collections held by NGB for other gene banks, are under common Nordic management and in the public domain”. The material in the active collection is available under the Standard Material Transfer Agreement (SMTA) of the International Treaty on Plant Genetic Resources for Food and Agriculture [9].

### 4.4. Ecogeographic Representativeness

Within-species genetic diversity is central for addressing future plant breeding and food security challenges such as an increasing human population and adaptation to climate change [47]. However, it is time-consuming and expensive to evaluate the relevant variation directly, especially across many species and large geographical areas. Ecogeographic analyses are based on available environmental data and predict the plants’ potential adaptation to defined growing conditions, and in this way predict their genetic adaptation [31]. It is a cost-effective method to get an idea of the adaptive genetic diversity of a wide range of species growing in different conditions. Theory predicts that wild plant species will become adapted to their local environment over time, and there are empirical studies suggesting that environmental and geographical data can be efficient in predicting patterns of genetic diversity [48]. However, more research is needed to confirm these results.

In the future, it would be valuable to evaluate the diversity of the target populations with genetic markers and/or anatomical traits, to be able to target collecting efforts to the most genetically distant and diverse populations. In addition, research efforts on the population structures and effective collecting approaches need to be performed in several species.

### 4.5. Links between Regional and National CWR Conservation

The complementary network for in situ conservation and ex situ gap analysis collection sites can be used as a basis for future conservation actions in Finland. Finnish CWR conservation has been addressed on both Nordic regional [23] and national levels [49]. For ex situ conservation, a common Nordic gene bank for seeds has been established since 1979 and is now part of NordGen, which also coordinates a number of working groups that form a network with experts dedicated to the conservation and use of genetic resources, including the recently established CWR working group. Such an established co-operative genetic resource conservation network does not yet exist within in situ conservation of CWR, even though the project-based Nordic CWR network has been active since 2015. With an established long-term in situ network, co-operation and knowledge exchange would be facilitated and national work could be complemented with analysis and optimisation on the Nordic level.

This article is based on national work taken forward in Finland in accordance with regional recommendations specified in the Nordic CWR project report [43] and primary steps were defined in the Nordic CWR conservation planning co-operation project. We aimed to find nationally important PAs for CWR genetic reserve sites, but many of the sites also have Nordic regional importance, being part of the proposed Nordic CWR conservation in situ site network [43]. The complementary PAs in both Finnish and Nordic analysis were the Archipelago Sea National Park, Koli National Park, Pallas-Ylläs National Park, Tammisaari and Hanko Archipelago, Perämeri Islands and Revonneva-Ruonneva conservation area.

The role of decision makers is essential in order to get CWR conservation implemented. The main future tasks are to implement active in situ conservation, including necessary monitoring and management actions; to establish and implement ex situ collecting strategies; and to enable access to CWR genetic resources for their sustainable utilization in plant breeding and research. These are key activities to assure long-term conservation and use [50]. More research is needed on CWR genetic diversity, conservation methods and the effect of climate change on CWR populations. Climate change poses a challenge to wild populations with shifts in vegetation in the future. Therefore, the impact of climate change on CWR populations needs to be looked into. Climate change scenarios can be used to predict distributional changes and to help plan conservation of CWR [39,51,52]. To achieve these, funds are needed as well as sharing of responsibilities and resources among agricultural and environmental actors. In addition, awareness raising regarding the importance of CWR, and their conservations needs is essential to assure future CWR conservation. Harmonization of the national priority lists and the Nordic list would make regional conservation efforts easier, as well as regionally complementary in situ sites and ex situ accessions.

### 4.6. National in Situ and Ex Situ Conservation Recommendations

The findings from the ex situ and in situ gap analyses combined with recommendations from previous Finnish CWR conservation reports [17,49] can form new national conservation recommendations. These include:

- The in situ sites (Table A2 and Table A3) from the gap analyses need to be inventoried to identify target populations for in situ conservation and plans should be made for inclusion of these CWR populations into protected area management and monitoring. Conservation efforts should first be focused on the top 10 sites, and ideally on all the 49 or 59 complementary sites, depending on whether the mainland Finland and Åland Islands or mainland Finland complementary network is selected for implementation.
- Co-operation between agricultural and nature management authorities is essential for the implementation of active in situ conservation of CWR taxa and for enabling their utilization.
- Seed collection for ex situ conservation should be conducted from the sites identified in the analyses (Table S4) both to ensure long-term backup of accessions conserved in situ and to provide access to the material. The seeds are planned to be stored in either NordGen or the Finnish national seedbank and backed up according to existing systems.
- Both the in situ and ex situ gap analyses should be re-done at suitable time intervals as more data are collected, new accessions are conserved ex situ and in situ conservation is established for targeted populations. Complementarity of CWR population conservation should take into account both national and Nordic levels so that the in situ sites and ex situ collections complement each other on a Nordic level.
- An active national CWR network of experts and stakeholders is needed to take the implementation of CWR conservation forward in Finland.

## Figures and Tables

**Table 1:** CWR priority species in in situ and ex situ conservation analysis. Only accessions with longitude and latitude data and which are stored long-term were included.

Priority Species in Ex Situ and In Situ Conservation Analyses	Ex Situ Accessions	Observations in the Wild	In Situ Complementarity Analysis	Ex Situ Analysis Representative-ness Analysis	Ex Situ Complementarity Analysis
*Allium schoenoprasum* L.	13	1619	x	x	x
*Armoracia rusticana* P. Gaertn., B. Mey. & Scherb.	0	707	x		x
*Asparagus officinalis* L.	0	67	x		x
*Barbarea stricta* Andrz.	0	3267	x		x
*Barbarea vulgaris* R. Br.	0	4862	x		x
*Brassica rapa* L.	4	2716	x	x	x
*Bromopsis inermis* (Leyss.) Holub	0	764	x		x
*Carum carvi* L.	4	4265	x	x	x
*Chenopodium ficifolium* Sm.	0	121	x		x
*Cichorium intybus* L.	0	80	x		x
*Corylus avellana* L.	0	1523	x		x
*Crambe maritima* L.	1	89	x		x
*Dactylis glomerata* L.	37	8454	x	x	x
*Diplotaxis muralis* (L.) DC.	0	5	x		x
*Diplotaxis tenuifolia* (L.) DC.	0	18	x		x
*Erucastrum gallicum* (Willd.) O. E. Schulz	0	54	x		x
*Festuca nigrescens* Lam.	0	39	x		x
*Festuca ovina* L.	21	14,094	x	x	x
*Festuca rubra* L.	56	13,238	x	x	x
*Festuca trachyphylla* (Hack.) Krajina	0	1436	x		x
*Fragaria moschata* Weston	0	899	x		x
*Fragaria vesca* L.	0	12,947	x		x
*Fragaria viridis* Weston	0	19	x		x
*Lactuca sibirica* (L.) Benth. ex Maxim.	0	538	x		x
*Lactuca tatarica* (L.) C. A. Mey.	0	14	x		x
*Lolium multiflorum* Lam.	0	407	x		x
*Lolium perenne* L.	0	1864	x		x
*Malus sylvestris* Mill.	5	157	x		
*Medicago lupulina* L.	0	440	x		x
*Medicago sativa* L.	0	209	x		x
*Mentha aquatica* L.	0	4921	x		x
*Mentha arvensis* L.	2	30	x		x
*Phalaroides arundinacea* (L.) Rauschert	60	8102	x	x	x
*Phleum nodosum* L.	0	30	x		x
*Phleum pratense* L.	208	14,318	x	x	x
*Poa alpina* L.	0	692	x		x
*Poa pratensis* L.	2	11,393	x	x	x
*Prunus spinosa* L.	0	35	x		x
*Ribes nigrum* L.	4	6286	x	x	x
*Ribes spicatum* E. Robson	1	4685	x	x	x
*Ribes uva-crispa* L.	0	1657	x		x
*Rubus arcticus* L.	0	8193	x		x
*Rubus caesius* L.	0	72	x		x
*Rubus chamaemorus* L.	0	9543	x		x
*Rubus idaeus* L.	0	16,782	x		x
*Schedonorus pratensis* (Huds.) P. Beauv.	15	6628	x	x	x
*Sinapis arvensis* L.	0	271	x		x
*Trifolium hybridum* L.	6	7773	x	x	x
*Trifolium pratense* L.	98	15,683	x	x	x
*Trifolium repens* L.	18	16,747	x	x	x
*Vaccinium microcarpum* (Turcz. ex Rupr.) Schmalh.	0	3651	x		x
*Vaccinium myrtillus* L.	1	21,672	x	x	x
*Vaccinium oxycoccos* L.	1	8365	x	x	x
*Vaccinium uliginosum* L.	1	15,127	x		x
*Vaccinium vitis-idaea* L.	1	22,714	x	x	x
*Vicia lathyroides* L.	0	15	x		x

## Data Availability

The data not included in the Supplementary Materials are available by request from the author.

## Supplementary Materials

The following supporting information can be downloaded at: https://www.mdpi.com/article/10.3390/plants12183313/s1. Table S1: Average values of variables in ELC categories; Table S2: Mainland Finland in situ complementary sites and species; Table S3: Mainland Finland and Åland Islands in situ complementary sites and species and Table S4: Ex situ gap analysis—complementary species/ELC in collecting sites.

## Appendix A

**Table A1:** The ex situ analysis of species-specific variables.

Code	Variables Selected	Unit	Source	*Allium schoenoprasum*	*Brassica rapa*	*Carum carvi*	*Dactylis glomerata*	*Festuca ovina*	*Festuca rubra*	*Phalaroides arundinacea*	*Phleum pratense*	*Poa pratensis*	*Ribes nigrum*	*Ribes spicatum*	*Schenodorus pratensis*	*Trifolium hybridum*	*Trifolium pratense*	*Trifolium repens*	*Vaccinium myrtillus*	*Vaccinium oxycoccus*	*Vaccinium vitis-idaea*
	**Bioclimatic**																				
**bio_1**	Annual average temperature	°C	Worldclim	x	x			x	x		x	x			x	x				x	x
**bio_2**	Average daytime temperature	°C	Worldclim			x				x			x						x		
**bio_3**	Isothermality	°C	Worldclim				x							x	x						
**bio_4**	Temperature seasonality		Worldclim				x	x	x			x	x			x	x	x			
**bio_5**	Maximum temperature for the warmest month	°C	Worldclim																		
**bio_6**	Minimum temperature for the coldest month	°C	Worldclim																		
**bio_7**	Annual temperature range	°C	Worldclim					x	x							x	x	x			
**bio_8**	Average temperature for the wettest quarter	°C	Worldclim			x						x									
**bio_9**	Average temperature for the driest quarter	°C	Worldclim	x							x				x	x					
**bio_10**	Average temperature for the hottest quarter	°C	Worldclim		x									x							
**bio_11**	Average temperature for the coldest quarter	°C	Worldclim				x												x	x	x
**bio_12**	Annual rainfall	°C	Worldclim							x											
**bio_13**	Rainfall during the wettest month	mm	Worldclim		x												x	x			
**bio_14**	Rainfall during the driest month	mm	Worldclim				x							x							
**bio_15**	Seasonality of rainfall	mm	Worldclim											x			x	x			
**bio_16**	Rainfall during the wettest quarter	mm	Worldclim	x						x	x		x		x				x	x	x
**bio_17**	Rainfall during the driest quarter	mm	Worldclim		x	x															
**bio_18**	Rainfall during the hottest quarter	mm	Worldclim							x	x		x						x	x	x
**bio_19**	Rainfall during the coldest quarter	mm	Worldclim	x		x		x	x			x									
	**Edaphic**																				
**ref_depth**	Soil depth	M	HWS database											x							
**t_bs**	Topsoil base saturation	%	HWS database	x																	
**t_ref_bulk**	Topsoil bulk density	kg/dm3	HWS database		x	x	x				x	x					x	x			
**t_caco3**	Topsoil calcium carbonate	% weight	HWS database							x					x				x	x	x
**t_cec_clay**	Topsoil clay cation exchange capacity in surface soil	cmol/kg	HWS database		x	x								x	x	x	x	x			
**t_cec_soil**	Topsoil cation exchange capacity in surface soil	cmol/kg	HWS database			x		x	x			x	x								
**t_clay**	Topsoil clay fraction	% weight	HWS database				x			x							x	x			
**t_gravel**	Topsoil gravel	% weight	HWS database			x		x	x				x		x						
**t_caso4**	Topsoil gypsum	% weight	HWS database				x														
**t_oc**	Topsoil organic carbon content	% weight	HWS database	x						x	x		x		x						
**t_ph_h2o**	Topsoil pH in a soil-water solution	−log(H+)	HWS database	x							x					x					
**t_ece**	Topsoil salinity	dS/m	HWS database				x							x		x			x	x	x
**t_sand**	Topsoil sand fraction	% weight	HWS database	x	x							x									
**t_silt**	Topsoil silt fraction	% weight	HWS database		x			x	x		x		x				x	x	x	x	x
**t_esp**	Topsoil sodicity	%	HWS database					x	x	x		x		x		x			x	x	x
	**Geophysic**																				
**aspect**	Elevation	NA	Worldclim	x	x	x	x	x	x	x	x	x	x	x	x	x	x	x	x	x	x
**alt**	Aspect	NA	NA	x	x	x	x	x	x	x	x	x	x	x	x	x	x	x	x	x	x
**eastness**	Eastness	NA	NA	x	x	x	x	x	x	x	x	x	x	x	x	x	x	x	x	x	x
**northness**	Northness	NA	NA	x	x	x	x	x	x	x	x	x	x	x	x	x	x	x	x	x	x

**Table A2:** Mainland Finland in situ complementary sites and species/ELC number (Site ID number provided by the World Database of Protected Areas).

Complementary Site	Site Id	Site Name	Number of Species/Elc Combinations
1	853	Oulangan kansallispuisto	76
2	852	Nuuksion kansallispuisto	63
3	867	Torronsuon kansallispuisto	32
4	824	Laajalahden luonnonsuojelualue	31
5	1960	Kaldoaivin erämaa	25
6	1404	Rahjan saaristo-Siiponjoen suisto	23
7	1021	Korouoma	21
8	59040	Sipoonkorven kansallispuisto	10
9	1662	Vaarajänkkä-Rovajänkkä	10
10	20625	Räyringin lehdot	9
11	62665	Losonvaara	9
12	1326	Preiviikinlahti	8
13	34361	Pallas-Yllästunturin kansallispuisto	7
14	64941	Laukkallion luonnonsuojelualue	5
15	28034	Martinselkonen	5
16	42162	Kolin kansallispuisto	4
17	93381	Itämäen luonnonsuojelualue	4
18	31313	Närängänvaara	4
19	30421	Huosianmaankallion lehtoalue	4
20	42204	Saanan lehtojensuojelualue	3
21	858	Pyhä-Häkin kansallispuisto	3
22	1353	Kellojärvi	3
23	868	Urho Kekkosen kansallispuisto	3
24	1453	Heinäjänkä-Karhuaapa-Kokonräme	3
25	43401	Päätyeenlahti	2
26	25870	Kummelbergen	2
27	25633	Aulangon luonnonsuojelualue	2
28	45483	Rauhanmaja	2
29	1331	Siikalahti	2
30	1610	Revonneva-Ruoneva	2
31	20805	Suomijärvi	2
32	42203	Liimanninkosken lehtojensuojelualue	2
33	24735	Lohjanjärven alueet	2
34	860	Riisitunturin kansallispuisto	2
35	928	Värriön luonnonpuisto	2
36	81833	Kutujoki	2
37	966	Issakka	2
38	83162	Kielajoen harju	1
39	26270	Lövkullauddenin lehtoalue-Lohjanjärvi	1
40	843	Isojärven kansallispuisto	1
41	83164	Järämä	1
42	889	Lohijoen lehtojensuojelualue	1
43	1149	Ruosmesuo-Hanhisuo	1
44	849	Lemmenjoen kansallispuisto	1
45	1512	Kiimingin lettoalue	1
46	83163	Kalddasjohka	1
47	45761	Valklamminsuo	1
48	47832	Ohkolanjokilaakso	1
49	1641	Suuripään alue-Saaranojan lehto	1
50	862	Saaristomeren kansallispuisto	1
51	1962	Käsivarren erämaa	1
52	89186	Pirnesoja	1
53	828	Martinselkosen luonnonsuojelualue	1
54	1403	Päijänteen keskiosa-Edessalo-Vaarunvuoret	1
55	93419	Sorsatunturin luonnonsuojelualue	1
56	35719	Kaalijoen ja Pyydysmäen taponlehtilehdot	1
57	92822	Hossan kansallispuisto	1
58	64798	Louhensuon luonnonsuojelualue	1
59	25750	Pisavaaran luonnonpuisto	1
60	27652	Patvinsuo	1
61	64792	Jylkkyvaaran luonnonsuojelualue	1
62	1647	Särkivaaran-Löyhkösen alue	1

**Table A3:** Mainland Finland and Åland Islands in situ complementary sites and species/ELC number (Site ID number provided by the World Database of Protected Areas).

Complementary Site	Site Id	Site Name	Number of Species/Elc Combinations
1	555543215	Tammisaaren ja Hangon saariston ja Pohjanpitäjänlahden merensuojelualueproteciton area	57
2	555580012	Uk-Puisto-Sompio-Kemihaara	51
3	555525183	Kiiminkijoki	42
4	555524483	Evon alue	23
5	902780	Oulangan kansallispuisto	12
6	555524301	Hyyppärän harjualue	9
7	555543221	Rahjan saaristo	7
8	555525477	Ounasjoki	6
9	902791	Torronsuon kansallispuisto	6
10	555580240	Etelä-Kuusamon metsät	5
11	555524496	Kukkiajärvi	4
12	555580002	Värriö	4
13	555525566	Västra Espholm	4
14	149649	Saanan lehtojensuojelualue	3
15	902757	Rääkkylän and Kiteen lintujärvet	3
16	555579976	Pallas-Ounastunturi	3
17	555543214	Saaristomeri	3
18	555525221	Pitkäsneva	3
19	555525292	Kinnussuo-Mustinsuo	3
20	555524242	Lohjanjärven alueet	2
21	555580065	Luvian saaristo	2
22	555525143	Lestijoki	2
23	555579982	Perämeren saaret	2
24	555524541	Liesjärvi	2
25	555579975	Käsivarren Erämaa	2
26	555525199	Paljakan metsät ja suot	2
27	555525515	Paistunturin erämaa	1
28	555580055	Ruissalon lehdot	1
29	555580018	Kaldoaivin Erämaa	1
30	388514	Kalkkikallion luonnonsuojelualue	1
31	555524330	Pomponrahka	1
32	555524355	Rekijokilaakso	1
33	555525358	Vasonniemi ja Pahalammenpuro	1
34	151068	Tornion linnustonsuojelualueet	1
35	555525516	Pulmankijärvi	1
36	555563330	Lehtola	1
37	902787	Siikalahti	1
38	555580272	Revonneva-Ruonneva	1
39	555525258	Kurimonkosken niityt	1
40	329930	Myllyharjun luonnonsuojelualue	1
41	555538774	Suomijärvi	1
42	555543217	Hailuoto, pohjoisranta	1
43	555589893	Riutan merenrantaniitty	1
44	555525372	Martinselkonen	1
45	555525208	Muhos-ja Poikajoen alueet	1
46	555580235	Valtavaara-Pyhävaara	1
47	555525507	Vaarajänkkä-Rovajänkkä	1
48	555525373	Jylkkyvaara ja Jylkynsuo	1
49	555580285	Juortanansalon alue	1
50	555538773	Viurilanlahti	1
51	555525576	Gloviken	1
52	555525530	Nåtö-Jungfruskär	1
