# Supplementary material for: Complementary Analysis and Implementation Plan for Conservation of Crop Wild Relatives in Finland
Source: Plants (Basel). 2023 Sep 19;12(18):3313. doi: 10.3390/plants12183313 (PMC10537885; doi:10.3390/plants12183313)
Supplement: Supplementary file 1 [file plants-12-03313-s001.zip › plants-2439733-supplementary.pdf]

SUPPLEMENTARY MATERIALS

Table S1. Average values of variables in ELC categories

Table S2. Mainland Finland *in situ* complementary sites and species

Table S3. Mainland Finland and Åland Islands *in situ* complementary sites and species

Table S4. *Ex situ* gap analysis - complementary species/ELC in collecting sites

Table S1. Average values of variables in ELC categories

| ELC category (zone) | Bio_12: average annual rainfall (mm) | Bio_7: annual average temp. range(°C) | Ref_depth: average elevation above sealevel (m) | Average eastness | Average northness | Average slope (degrees of the land surface) | Latitude (decimal degrees) | T_oc : average organic carbon content in surface soil (% weight) | t_ph_h2o: average pH in soil water solution in surface soil (log(H+)) | ref_depth: average depth of the soil unit (m) |
|---|---|---|---|---|---|---|---|---|---|---|
| 1 | 520 | 3.62 | 119 | 0.000 | 0.066 | 189.782 | 65.216804 | 33.630 | 4.300 | 100 |
| 2 | 535 | 3.44 | 13 | 0.065 | 0.059 | 166.718 | 63.850137 | 1.400 | 7.500 | 10 |
| 3 | 522 | 3.54 | 95 | 0.000 | 0.065 | 194.744 | 64.541804 | 2.180 | 4.400 | 100 |
| 4 | 514 | 3.72 | 227 | 0.000 | 0.041 | 180.000 | 67.116804 | 33.630 | 4.300 | 100 |
| 5 | 486 | 3.69 | 261 | 0.000 | 0.039 | 175.236 | 68.200137 | 2.180 | 4.400 | 100 |
| 6 | 603 | 3.43 | 157 | 0.013 | 0.047 | 181.548 | 62.883470 | 33.630 | 4.300 | 100 |
| 7 | 621 | 3.12 | 39 | 0.017 | 0.030 | 180.000 | 60.575137 | 0.960 | 7.300 | 100 |
| 8 | 618 | 3.37 | 114 | 0.000 | 0.060 | 178.668 | 62.300137 | 2.180 | 4.400 | 100 |
| 9 | 595 | 3.65 | 258 | 0.024 | 0.022 | 186.115 | 65.166804 | 33.630 | 4.300 | 100 |
| 10 | 522 | 3.28 | 266 | 0.013 | 0.008 | 170.489 | 69.125137 | 2.180 | 4.400 | 100 |

Table S2. Mainland Finland *in situ* complementary sites and species. (Site ID number provided by the World Database of Protected Areas)

| COMPLEMENTARY SITE | SITE ID | COMPLEMENTARY SPECIES | ELC CATEGORY | SITE NAME |
|---|---|---|---|---|
| 1 | 853 | Barbarea stricta | 10 | Oulanka National Park |
| 1 | 853 | Barbarea stricta | 13 | Oulanka National Park |
| 1 | 853 | Dactylis glomerata | 13 | Oulanka National Park |
| 1 | 853 | Festuca ovina | 9 | Oulanka National Park |
| 1 | 853 | Festuca ovina | 10 | Oulanka National Park |
| 1 | 853 | Festuca ovina | 12 | Oulanka National Park |
| 1 | 853 | Festuca ovina | 13 | Oulanka National Park |
| 1 | 853 | Festuca ovina | 15 | Oulanka National Park |
| 1 | 853 | Festuca rubra | 10 | Oulanka National Park |
| 1 | 853 | Festuca rubra | 13 | Oulanka National Park |
| 1 | 853 | Festuca rubra | 15 | Oulanka National Park |
| 1 | 853 | Festuca trachyphylla | 10 | Oulanka National Park |
| 1 | 853 | Fragaria vesca | 10 | Oulanka National Park |
| 1 | 853 | Fragaria vesca | 13 | Oulanka National Park |
| 1 | 853 | Lolium perenne | 13 | Oulanka National Park |
| 1 | 853 | Mentha arvensis | 15 | Oulanka National Park |
| 1 | 853 | Phalaroides arundinacea | 9 | Oulanka National Park |
| 1 | 853 | Phalaroides arundinacea | 10 | Oulanka National Park |
| 1 | 853 | Phalaroides arundinacea | 12 | Oulanka National Park |
| 1 | 853 | Phalaroides arundinacea | 13 | Oulanka National Park |
| 1 | 853 | Phalaroides arundinacea | 15 | Oulanka National Park |
| 1 | 853 | Phleum pratense | 10 | Oulanka National Park |
| 1 | 853 | Phleum pratense | 13 | Oulanka National Park |
| 1 | 853 | Phleum pratense | 15 | Oulanka National Park |
| 1 | 853 | Poa alpina | 13 | Oulanka National Park |
| 1 | 853 | Poa pratensis | 13 | Oulanka National Park |
| 1 | 853 | Poa pratensis | 15 | Oulanka National Park |
| 1 | 853 | Ribes spicatum | 10 | Oulanka National Park |
| 1 | 853 | Ribes spicatum | 13 | Oulanka National Park |
| 1 | 853 | Ribes spicatum | 15 | Oulanka National Park |
| 1 | 853 | Rubus arcticus | 9 | Oulanka National Park |
| 1 | 853 | Rubus arcticus | 10 | Oulanka National Park |
| 1 | 853 | Rubus arcticus | 12 | Oulanka National Park |
| 1 | 853 | Rubus arcticus | 13 | Oulanka National Park |
| 1 | 853 | Rubus arcticus | 15 | Oulanka National Park |
| 1 | 853 | Rubus chamaemorus | 9 | Oulanka National Park |
| 1 | 853 | Rubus chamaemorus | 10 | Oulanka National Park |
| 1 | 853 | Rubus chamaemorus | 12 | Oulanka National Park |
| 1 | 853 | Rubus chamaemorus | 13 | Oulanka National Park |
| 1 | 853 | Rubus chamaemorus | 15 | Oulanka National Park |

| | | | | |
|---|---|---|---|---|
| 1 | 853 | Rubus idaeus | 10 | Oulanka National Park |
| **COMPLEMENTARY SITE** | **SITE ID** | **COMPLEMENTARY SPECIES** | **ELC CATEGORY** | **SITE NAME** |
| 1 | 853 | Rubus idaeus | 13 | Oulanka National Park |
| 1 | 853 | Rubus idaeus | 15 | Oulanka National Park |
| 1 | 853 | Schedonorus pratensis | 10 | Oulanka National Park |
| 1 | 853 | Trifolium pratense | 10 | Oulanka National Park |
| 1 | 853 | Trifolium pratense | 13 | Oulanka National Park |
| 1 | 853 | Trifolium repens | 10 | Oulanka National Park |
| 1 | 853 | Trifolium repens | 13 | Oulanka National Park |
| 1 | 853 | Trifolium repens | 15 | Oulanka National Park |
| 1 | 853 | Vaccinium microcarpum | 9 | Oulanka National Park |
| 1 | 853 | Vaccinium microcarpum | 10 | Oulanka National Park |
| 1 | 853 | Vaccinium microcarpum | 12 | Oulanka National Park |
| 1 | 853 | Vaccinium microcarpum | 13 | Oulanka National Park |
| 1 | 853 | Vaccinium microcarpum | 15 | Oulanka National Park |
| 1 | 853 | Vaccinium myrtillus | 9 | Oulanka National Park |
| 1 | 853 | Vaccinium myrtillus | 10 | Oulanka National Park |
| 1 | 853 | Vaccinium myrtillus | 12 | Oulanka National Park |
| 1 | 853 | Vaccinium myrtillus | 13 | Oulanka National Park |
| 1 | 853 | Vaccinium myrtillus | 15 | Oulanka National Park |
| 1 | 853 | Vaccinium oxycoccos | 10 | Oulanka National Park |
| 1 | 853 | Vaccinium oxycoccos | 12 | Oulanka National Park |
| 1 | 853 | Vaccinium oxycoccos | 13 | Oulanka National Park |
| 1 | 853 | Vaccinium oxycoccos | 15 | Oulanka National Park |
| 1 | 853 | Vaccinium uliginosum | 9 | Oulanka National Park |
| 1 | 853 | Vaccinium uliginosum | 10 | Oulanka National Park |
| 1 | 853 | Vaccinium uliginosum | 12 | Oulanka National Park |
| 1 | 853 | Vaccinium uliginosum | 13 | Oulanka National Park |
| 1 | 853 | Vaccinium uliginosum | 15 | Oulanka National Park |
| 1 | 853 | Vaccinium vitis-idaea | 9 | Oulanka National Park |
| 1 | 853 | Vaccinium vitis-idaea | 10 | Oulanka National Park |
| 1 | 853 | Vaccinium vitis-idaea | 12 | Oulanka National Park |
| 1 | 853 | Vaccinium vitis-idaea | 13 | Oulanka National Park |
| 1 | 853 | Vaccinium vitis-idaea | 15 | Oulanka National Park |
| 2 | 852 | Barbarea vulgaris | 2 | Nuuksio National park |
| 2 | 852 | Barbarea vulgaris | 3 | Nuuksio National park |
| 2 | 852 | Barbarea vulgaris | 7 | Nuuksio National park |
| 2 | 852 | Barbarea vulgaris | 8 | Nuuksio National park |
| 2 | 852 | Carum carvi | 3 | Nuuksio National park |
| 2 | 852 | Corylus avellana | 7 | Nuuksio National park |
| 2 | 852 | Dactylis glomerata | 2 | Nuuksio National park |
| 2 | 852 | Dactylis glomerata | 7 | Nuuksio National park |
| 2 | 852 | Festuca ovina | 2 | Nuuksio National park |
| 2 | 852 | Festuca ovina | 7 | Nuuksio National park |
| 2 | 852 | Festuca rubra | 2 | Nuuksio National park |
| 2 | 852 | Festuca rubra | 7 | Nuuksio National park |

| | | | | |
|---|---|---|---|---|
| 2 | 852 | Fragaria vesca | 2 | Nuuksio National park |
| **COMPLEMENTARY SITE** | **SITE ID** | **COMPLEMENTARY SPECIES** | **ELC CATEGORY** | **SITE NAME** |
| 2 | 852 | Fragaria vesca | 7 | Nuuksio National park |
| 2 | 852 | Lolium perenne | 7 | Nuuksio National park |
| 2 | 852 | Mentha arvensis | 2 | Nuuksio National park |
| 2 | 852 | Mentha arvensis | 7 | Nuuksio National park |
| 2 | 852 | Phleum pratense | 2 | Nuuksio National park |
| 2 | 852 | Phleum pratense | 3 | Nuuksio National park |
| 2 | 852 | Phleum pratense | 7 | Nuuksio National park |
| 2 | 852 | Phleum pratense | 8 | Nuuksio National park |
| 2 | 852 | Poa pratensis | 2 | Nuuksio National park |
| 2 | 852 | Poa pratensis | 3 | Nuuksio National park |
| 2 | 852 | Poa pratensis | 7 | Nuuksio National park |
| 2 | 852 | Ribes nigrum | 2 | Nuuksio National park |
| 2 | 852 | Ribes nigrum | 7 | Nuuksio National park |
| 2 | 852 | Ribes spicatum | 2 | Nuuksio National park |
| 2 | 852 | Rubus chamaemorus | 2 | Nuuksio National park |
| 2 | 852 | Rubus chamaemorus | 3 | Nuuksio National park |
| 2 | 852 | Rubus chamaemorus | 7 | Nuuksio National park |
| 2 | 852 | Rubus idaeus | 2 | Nuuksio National park |
| 2 | 852 | Rubus idaeus | 3 | Nuuksio National park |
| 2 | 852 | Rubus idaeus | 7 | Nuuksio National park |
| 2 | 852 | Rubus idaeus | 8 | Nuuksio National park |
| 2 | 852 | Schedonorus pratensis | 2 | Nuuksio National park |
| 2 | 852 | Schedonorus pratensis | 7 | Nuuksio National park |
| 2 | 852 | Trifolium hybridum | 2 | Nuuksio National park |
| 2 | 852 | Trifolium hybridum | 7 | Nuuksio National park |
| 2 | 852 | Trifolium pratense | 2 | Nuuksio National park |
| 2 | 852 | Trifolium pratense | 3 | Nuuksio National park |
| 2 | 852 | Trifolium pratense | 7 | Nuuksio National park |
| 2 | 852 | Trifolium repens | 2 | Nuuksio National park |
| 2 | 852 | Trifolium repens | 3 | Nuuksio National park |
| 2 | 852 | Trifolium repens | 7 | Nuuksio National park |
| 2 | 852 | Trifolium repens | 8 | Nuuksio National park |
| 2 | 852 | Vaccinium microcarpum | 2 | Nuuksio National park |
| 2 | 852 | Vaccinium microcarpum | 7 | Nuuksio National park |
| 2 | 852 | Vaccinium myrtillus | 2 | Nuuksio National park |
| 2 | 852 | Vaccinium myrtillus | 3 | Nuuksio National park |
| 2 | 852 | Vaccinium myrtillus | 7 | Nuuksio National park |
| 2 | 852 | Vaccinium myrtillus | 8 | Nuuksio National park |
| 2 | 852 | Vaccinium oxycoccos | 2 | Nuuksio National park |
| 2 | 852 | Vaccinium oxycoccos | 3 | Nuuksio National park |
| 2 | 852 | Vaccinium oxycoccos | 7 | Nuuksio National park |
| 2 | 852 | Vaccinium uliginosum | 2 | Nuuksio National park |
| 2 | 852 | Vaccinium uliginosum | 3 | Nuuksio National park |
| 2 | 852 | Vaccinium uliginosum | 7 | Nuuksio National park |

| | | | | |
|---|---|---|---|---|
| 2 | 852 | Vaccinium uliginosum | 8 | Nuuksio National park |
| **COMPLEMENTARY SITE** | **SITE ID** | **COMPLEMENTARY SPECIES** | **ELC CATEGORY** | **SITE NAME** |
| 2 | 852 | Vaccinium vitis-idaea | 2 | Nuuksio National park |
| 2 | 852 | Vaccinium vitis-idaea | 3 | Nuuksio National park |
| 2 | 852 | Vaccinium vitis-idaea | 7 | Nuuksio National park |
| 2 | 852 | Vaccinium vitis-idaea | 8 | Nuuksio National park |
| 3 | 867 | Barbarea vulgaris | 6 | Torronsuo National Park |
| 3 | 867 | Brassica rapa | 6 | Torronsuo National Park |
| 3 | 867 | Carum carvi | 6 | Torronsuo National Park |
| 3 | 867 | Festuca ovina | 6 | Torronsuo National Park |
| 3 | 867 | Festuca rubra | 1 | Torronsuo National Park |
| 3 | 867 | Festuca rubra | 6 | Torronsuo National Park |
| 3 | 867 | Fragaria vesca | 1 | Torronsuo National Park |
| 3 | 867 | Fragaria vesca | 6 | Torronsuo National Park |
| 3 | 867 | Phleum pratense | 1 | Torronsuo National Park |
| 3 | 867 | Phleum pratense | 6 | Torronsuo National Park |
| 3 | 867 | Poa pratensis | 1 | Torronsuo National Park |
| 3 | 867 | Poa pratensis | 6 | Torronsuo National Park |
| 3 | 867 | Ribes nigrum | 1 | Torronsuo National Park |
| 3 | 867 | Ribes nigrum | 6 | Torronsuo National Park |
| 3 | 867 | Rubus arcticus | 6 | Torronsuo National Park |
| 3 | 867 | Rubus chamaemorus | 1 | Torronsuo National Park |
| 3 | 867 | Rubus chamaemorus | 6 | Torronsuo National Park |
| 3 | 867 | Rubus idaeus | 1 | Torronsuo National Park |
| 3 | 867 | Rubus idaeus | 6 | Torronsuo National Park |
| 3 | 867 | Schedonorus pratensis | 1 | Torronsuo National Park |
| 3 | 867 | Trifolium hybridum | 6 | Torronsuo National Park |
| 3 | 867 | Trifolium pratense | 6 | Torronsuo National Park |
| 3 | 867 | Trifolium repens | 6 | Torronsuo National Park |
| 3 | 867 | Vaccinium microcarpum | 6 | Torronsuo National Park |
| 3 | 867 | Vaccinium myrtillus | 1 | Torronsuo National Park |
| 3 | 867 | Vaccinium myrtillus | 6 | Torronsuo National Park |
| 3 | 867 | Vaccinium oxycoccos | 1 | Torronsuo National Park |
| 3 | 867 | Vaccinium oxycoccos | 6 | Torronsuo National Park |
| 3 | 867 | Vaccinium uliginosum | 1 | Torronsuo National Park |
| 3 | 867 | Vaccinium uliginosum | 6 | Torronsuo National Park |
| 3 | 867 | Vaccinium vitis-idaea | 1 | Torronsuo National Park |
| 3 | 867 | Vaccinium vitis-idaea | 6 | Torronsuo National Park |
| 4 | 824 | Allium schoenoprasum | 3 | Laajalahti conservation area |
| 4 | 824 | Armoracia rusticana | 3 | Laajalahti conservation area |
| 4 | 824 | Barbarea stricta | 3 | Laajalahti conservation area |
| 4 | 824 | Barbarea stricta | 8 | Laajalahti conservation area |
| 4 | 824 | Bromopsis inermis | 8 | Laajalahti conservation area |
| 4 | 824 | Carum carvi | 8 | Laajalahti conservation area |
| 4 | 824 | Corylus avellana | 3 | Laajalahti conservation area |
| 4 | 824 | Corylus avellana | 8 | Laajalahti conservation area |

| | | | | |
|---|---|---|---|---|
| 4 | 824 | Dactylis glomerata | 3 | Laajalahti conservation area |
| **COMPLEMENTARY SITE** | **SITE ID** | **COMPLEMENTARY SPECIES** | **ELC CATEGORY** | **SITE NAME** |
| 4 | 824 | Dactylis glomerata | 8 | Laajalahti conservation area |
| 4 | 824 | Festuca ovina | 3 | Laajalahti conservation area |
| 4 | 824 | Festuca ovina | 8 | Laajalahti conservation area |
| 4 | 824 | Festuca rubra | 3 | Laajalahti conservation area |
| 4 | 824 | Festuca rubra | 8 | Laajalahti conservation area |
| 4 | 824 | Festuca trachyphylla | 3 | Laajalahti conservation area |
| 4 | 824 | Festuca trachyphylla | 8 | Laajalahti conservation area |
| 4 | 824 | Fragaria vesca | 3 | Laajalahti conservation area |
| 4 | 824 | Fragaria vesca | 8 | Laajalahti conservation area |
| 4 | 824 | Lolium perenne | 8 | Laajalahti conservation area |
| 4 | 824 | Phalaroides arundinacea | 3 | Laajalahti conservation area |
| 4 | 824 | Phalaroides arundinacea | 8 | Laajalahti conservation area |
| 4 | 824 | Poa pratensis | 8 | Laajalahti conservation area |
| 4 | 824 | Ribes nigrum | 3 | Laajalahti conservation area |
| 4 | 824 | Ribes nigrum | 8 | Laajalahti conservation area |
| 4 | 824 | Ribes spicatum | 8 | Laajalahti conservation area |
| 4 | 824 | Ribes uva-crispa | 3 | Laajalahti conservation area |
| 4 | 824 | Ribes uva-crispa | 8 | Laajalahti conservation area |
| 4 | 824 | Schedonorus pratensis | 8 | Laajalahti conservation area |
| 4 | 824 | Trifolium hybridum | 3 | Laajalahti conservation area |
| 4 | 824 | Trifolium hybridum | 8 | Laajalahti conservation area |
| 4 | 824 | Trifolium pratense | 8 | Laajalahti conservation area |
| 5 | 1960 | Allium schoenoprasum | 5 | Kaldoaivi Wilderness Area |
| 5 | 1960 | Barbarea stricta | 2 | Kaldoaivi Wilderness Area |
| 5 | 1960 | Festuca ovina | 4 | Kaldoaivi Wilderness Area |
| 5 | 1960 | Festuca ovina | 5 | Kaldoaivi Wilderness Area |
| 5 | 1960 | Festuca rubra | 4 | Kaldoaivi Wilderness Area |
| 5 | 1960 | Festuca rubra | 5 | Kaldoaivi Wilderness Area |
| 5 | 1960 | Fragaria vesca | 5 | Kaldoaivi Wilderness Area |
| 5 | 1960 | Poa alpina | 4 | Kaldoaivi Wilderness Area |
| 5 | 1960 | Poa alpina | 5 | Kaldoaivi Wilderness Area |
| 5 | 1960 | Poa pratensis | 4 | Kaldoaivi Wilderness Area |
| 5 | 1960 | Poa pratensis | 5 | Kaldoaivi Wilderness Area |
| 5 | 1960 | Rubus arcticus | 2 | Kaldoaivi Wilderness Area |
| 5 | 1960 | Rubus arcticus | 5 | Kaldoaivi Wilderness Area |
| 5 | 1960 | Rubus chamaemorus | 4 | Kaldoaivi Wilderness Area |
| 5 | 1960 | Rubus chamaemorus | 5 | Kaldoaivi Wilderness Area |
| 5 | 1960 | Vaccinium microcarpum | 4 | Kaldoaivi Wilderness Area |
| 5 | 1960 | Vaccinium microcarpum | 5 | Kaldoaivi Wilderness Area |
| 5 | 1960 | Vaccinium myrtillus | 4 | Kaldoaivi Wilderness Area |
| 5 | 1960 | Vaccinium myrtillus | 5 | Kaldoaivi Wilderness Area |
| 5 | 1960 | Vaccinium oxycoccos | 4 | Kaldoaivi Wilderness Area |
| 5 | 1960 | Vaccinium oxycoccos | 5 | Kaldoaivi Wilderness Area |
| 5 | 1960 | Vaccinium uliginosum | 4 | Kaldoaivi Wilderness Area |

| | | | | |
|---|---|---|---|---|
| 5 | 1960 | Vaccinium uliginosum | 5 | Kaldoaivi Wilderness Area |
| **COMPLEMENTARY SITE** | **SITE ID** | **COMPLEMENTARY SPECIES** | **ELC CATEGORY** | **SITE NAME** |
| 5 | 1960 | Vaccinium vitis-idaea | 4 | Kaldoaivi Wilderness Area |
| 5 | 1960 | Vaccinium vitis-idaea | 5 | Kaldoaivi Wilderness Area |
| 6 | 1404 | Festuca rubra | 11 | Rahja Archipelago |
| 6 | 1404 | Festuca rubra | 16 | Rahja Archipelago |
| 6 | 1404 | Fragaria vesca | 16 | Rahja Archipelago |
| 6 | 1404 | Phalaroides arundinacea | 11 | Rahja Archipelago |
| 6 | 1404 | Phalaroides arundinacea | 16 | Rahja Archipelago |
| 6 | 1404 | Poa pratensis | 11 | Rahja Archipelago |
| 6 | 1404 | Ribes nigrum | 11 | Rahja Archipelago |
| 6 | 1404 | Ribes spicatum | 11 | Rahja Archipelago |
| 6 | 1404 | Rubus arcticus | 11 | Rahja Archipelago |
| 6 | 1404 | Rubus arcticus | 16 | Rahja Archipelago |
| 6 | 1404 | Rubus chamaemorus | 11 | Rahja Archipelago |
| 6 | 1404 | Rubus chamaemorus | 16 | Rahja Archipelago |
| 6 | 1404 | Rubus idaeus | 11 | Rahja Archipelago |
| 6 | 1404 | Rubus idaeus | 16 | Rahja Archipelago |
| 6 | 1404 | Trifolium pratense | 11 | Rahja Archipelago |
| 6 | 1404 | Trifolium repens | 11 | Rahja Archipelago |
| 6 | 1404 | Vaccinium myrtillus | 11 | Rahja Archipelago |
| 6 | 1404 | Vaccinium myrtillus | 16 | Rahja Archipelago |
| 6 | 1404 | Vaccinium oxycoccos | 16 | Rahja Archipelago |
| 6 | 1404 | Vaccinium uliginosum | 11 | Rahja Archipelago |
| 6 | 1404 | Vaccinium uliginosum | 16 | Rahja Archipelago |
| 6 | 1404 | Vaccinium vitis-idaea | 11 | Rahja Archipelago |
| 6 | 1404 | Vaccinium vitis-idaea | 16 | Rahja Archipelago |
| 7 | 1021 | Festuca ovina | 14 | Korouoma Nature Reserve |
| 7 | 1021 | Festuca rubra | 12 | Korouoma Nature Reserve |
| 7 | 1021 | Festuca rubra | 14 | Korouoma Nature Reserve |
| 7 | 1021 | Fragaria vesca | 9 | Korouoma Nature Reserve |
| 7 | 1021 | Phalaroides arundinacea | 14 | Korouoma Nature Reserve |
| 7 | 1021 | Poa pratensis | 14 | Korouoma Nature Reserve |
| 7 | 1021 | Ribes nigrum | 12 | Korouoma Nature Reserve |
| 7 | 1021 | Ribes nigrum | 14 | Korouoma Nature Reserve |
| 7 | 1021 | Ribes spicatum | 9 | Korouoma Nature Reserve |
| 7 | 1021 | Ribes spicatum | 12 | Korouoma Nature Reserve |
| 7 | 1021 | Ribes spicatum | 14 | Korouoma Nature Reserve |
| 7 | 1021 | Rubus arcticus | 14 | Korouoma Nature Reserve |
| 7 | 1021 | Rubus chamaemorus | 14 | Korouoma Nature Reserve |
| 7 | 1021 | Rubus idaeus | 9 | Korouoma Nature Reserve |
| 7 | 1021 | Rubus idaeus | 12 | Korouoma Nature Reserve |
| 7 | 1021 | Rubus idaeus | 14 | Korouoma Nature Reserve |
| 7 | 1021 | Trifolium repens | 12 | Korouoma Nature Reserve |
| 7 | 1021 | Vaccinium myrtillus | 14 | Korouoma Nature Reserve |
| 7 | 1021 | Vaccinium oxycoccos | 14 | Korouoma Nature Reserve |

| | | | | |
|---|---|---|---|---|
| 7 | 1021 | Vaccinium uliginosum | 14 | Korouoma Nature Reserve |
| **COMPLEMENTARY SITE** | **SITE ID** | **COMPLEMENTARY SPECIES** | **ELC CATEGORY** | **SITE NAME** |
| 7 | 1021 | Vaccinium vitis-idaea | 14 | Korouoma Nature Reserve |
| 8 | 1662 | Festuca rubra | 9 | Vaarajänkkä-Rovajänkkä |
| 8 | 1662 | Fragaria vesca | 14 | Vaarajänkkä-Rovajänkkä |
| 8 | 1662 | Phleum pratense | 9 | Vaarajänkkä-Rovajänkkä |
| 8 | 1662 | Poa pratensis | 9 | Vaarajänkkä-Rovajänkkä |
| 8 | 1662 | Trifolium hybridum | 9 | Vaarajänkkä-Rovajänkkä |
| 8 | 1662 | Trifolium pratense | 9 | Vaarajänkkä-Rovajänkkä |
| 8 | 1662 | Trifolium pratense | 14 | Vaarajänkkä-Rovajänkkä |
| 8 | 1662 | Trifolium repens | 9 | Vaarajänkkä-Rovajänkkä |
| 8 | 1662 | Trifolium repens | 14 | Vaarajänkkä-Rovajänkkä |
| 8 | 1662 | Vaccinium oxycoccos | 9 | Vaarajänkkä-Rovajänkkä |
| 9 | 59040 | Barbarea stricta | 7 | Sipoonkorpi National Park |
| 9 | 59040 | Brassica rapa | 7 | Sipoonkorpi National Park |
| 9 | 59040 | Carum carvi | 2 | Sipoonkorpi National Park |
| 9 | 59040 | Carum carvi | 7 | Sipoonkorpi National Park |
| 9 | 59040 | Corylus avellana | 2 | Sipoonkorpi National Park |
| 9 | 59040 | Phalaroides arundinacea | 2 | Sipoonkorpi National Park |
| 9 | 59040 | Phalaroides arundinacea | 7 | Sipoonkorpi National Park |
| 9 | 59040 | Ribes spicatum | 7 | Sipoonkorpi National Park |
| 10 | 20625 | Barbarea stricta | 1 | Räyrinki |
| 10 | 20625 | Festuca ovina | 1 | Räyrinki |
| 10 | 20625 | Phalaroides arundinacea | 1 | Räyrinki |
| 10 | 20625 | Ribes uva-crispa | 1 | Räyrinki |
| 10 | 20625 | Rubus arcticus | 1 | Räyrinki |
| 10 | 20625 | Trifolium pratense | 1 | Räyrinki |
| 10 | 20625 | Trifolium repens | 1 | Räyrinki |
| 10 | 20625 | Vaccinium microcarpum | 1 | Räyrinki |
| 11 | 62665 | Barbarea vulgaris | 5 | Losonvaara |
| 11 | 62665 | Carum carvi | 5 | Losonvaara |
| 11 | 62665 | Dactylis glomerata | 5 | Losonvaara |
| 11 | 62665 | Phleum pratense | 5 | Losonvaara |
| 11 | 62665 | Ribes spicatum | 5 | Losonvaara |
| 11 | 62665 | Rubus idaeus | 5 | Losonvaara |
| 11 | 62665 | Trifolium hybridum | 5 | Losonvaara |
| 11 | 62665 | Trifolium pratense | 5 | Losonvaara |
| 11 | 62665 | Trifolium repens | 5 | Losonvaara |
| 12 | 1326 | Brassica rapa | 3 | Preiviikinlahti - Yyteri |
| 12 | 1326 | Bromopsis inermis | 3 | Preiviikinlahti - Yyteri |
| 12 | 1326 | Medicago sativa | 3 | Preiviikinlahti - Yyteri |
| 12 | 1326 | Mentha arvensis | 3 | Preiviikinlahti - Yyteri |
| 12 | 1326 | Ribes spicatum | 3 | Preiviikinlahti - Yyteri |
| 12 | 1326 | Rubus arcticus | 3 | Preiviikinlahti - Yyteri |
| 12 | 1326 | Schedonorus pratensis | 3 | Preiviikinlahti - Yyteri |
| 12 | 1326 | Vaccinium microcarpum | 3 | Preiviikinlahti - Yyteri |

| | | | | |
|---|---|---|---|---|
| 13 | 34361 | Barbarea vulgaris | 13 | Pallas-Yllästunturi National Park |
| **COMPLEMENTARY SITE** | **SITE ID** | **COMPLEMENTARY SPECIES** | **ELC CATEGORY** | **SITE NAME** |
| 13 | 34361 | Carum carvi | 13 | Pallas-Yllästunturi National Park |
| 13 | 34361 | Phleum pratense | 12 | Pallas-Yllästunturi National Park |
| 13 | 34361 | Poa alpina | 12 | Pallas-Yllästunturi National Park |
| 13 | 34361 | Poa pratensis | 12 | Pallas-Yllästunturi National Park |
| 13 | 34361 | Schedonorus pratensis | 13 | Pallas-Yllästunturi National Park |
| 13 | 34361 | Trifolium hybridum | 13 | Pallas-Yllästunturi National Park |
| 14 | 64941 | Allium schoenoprasum | 8 | Laukkallio conservation area |
| 14 | 64941 | Rubus arcticus | 8 | Laukkallio conservation area |
| 14 | 64941 | Rubus chamaemorus | 8 | Laukkallio conservation area |
| 14 | 64941 | Vaccinium microcarpum | 8 | Laukkallio conservation area |
| 14 | 64941 | Vaccinium oxycoccos | 8 | Laukkallio conservation area |
| 15 | 28034 | Bromopsis inermis | 14 | Martinselkonen nature reserve |
| 15 | 28034 | Carum carvi | 14 | Martinselkonen nature reserve |
| 15 | 28034 | Dactylis glomerata | 14 | Martinselkonen nature reserve |
| 15 | 28034 | Phleum pratense | 14 | Martinselkonen nature reserve |
| 15 | 28034 | Vaccinium microcarpum | 14 | Martinselkonen nature reserve |
| 16 | 42162 | Brassica rapa | 2 | Koli National Park |
| 16 | 42162 | Fragaria moschata | 2 | Koli National Park |
| 16 | 42162 | Ribes uva-crispa | 7 | Koli National Park |
| 16 | 42162 | Rubus arcticus | 7 | Koli National Park |
| 17 | 93381 | Barbarea stricta | 15 | Itämäki conservation area |
| 17 | 93381 | Carum carvi | 15 | Itämäki conservation area |
| 17 | 93381 | Fragaria vesca | 15 | Itämäki conservation area |
| 17 | 93381 | Trifolium pratense | 15 | Itämäki conservation area |
| 18 | 31313 | Carum carvi | 12 | Näränkävaara |
| 18 | 31313 | Trifolium pratense | 4 | Näränkävaara |
| 18 | 31313 | Trifolium pratense | 12 | Näränkävaara |
| 18 | 31313 | Trifolium repens | 4 | Näränkävaara |
| 19 | 30421 | Festuca trachyphylla | 6 | Huosianmaankallio |
| 19 | 30421 | Mentha arvensis | 6 | Huosianmaankallio |
| 19 | 30421 | Phalaroides arundinacea | 6 | Huosianmaankallio |
| 19 | 30421 | Schedonorus pratensis | 6 | Huosianmaankallio |
| 20 | 42204 | Barbarea stricta | 5 | Saana |
| 20 | 42204 | Festuca nigrescens | 5 | Saana |
| 20 | 42204 | Lolium perenne | 5 | Saana |
| 21 | 858 | Carum carvi | 1 | Pyhä-Häkki National Park |
| 21 | 858 | Ribes spicatum | 1 | Pyhä-Häkki National Park |
| 21 | 858 | Trifolium hybridum | 1 | Pyhä-Häkki National Park |
| 22 | 1353 | Dactylis glomerata | 10 | Kellojärvi |
| 22 | 1353 | Mentha arvensis | 10 | Kellojärvi |
| 22 | 1353 | Poa pratensis | 10 | Kellojärvi |
| 23 | 868 | Lactuca sibirica | 12 | Urho Kekkonen National Park |
| 23 | 868 | Lactuca sibirica | 13 | Urho Kekkonen National Park |
| 23 | 868 | Ribes nigrum | 13 | Urho Kekkonen National Park |

| COMPLEMENTARY SITE | SITE ID | COMPLEMENTARY SPECIES | ELC CATEGORY | SITE NAME |
|---|---|---|---|---|
| 24 | 1453 | Lactuca sibirica | 9 | Heinäjänkä-Karhuaapa-Kokonräme |
| 24 | 1453 | Lactuca sibirica | 14 | Heinäjänkä-Karhuaapa-Kokonräme |
| 24 | 1453 | Ribes nigrum | 15 | Heinäjänkä-Karhuaapa-Kokonräme |
| 25 | 26270 | Brassica rapa | 8 | Kummelbergen |
| 25 | 25870 | Mentha arvensis | 8 | Kummelbergen |
| 26 | 25870 | Lolium perenne | 2 | Aulanko conservation area |
| 26 | 25633 | Ribes uva-crispa | 2 | Aulanko conservation area |
| 27 | 25633 | Barbarea vulgaris | 1 | Rauhanmaja |
| 27 | 45483 | Fragaria moschata | 1 | Rauhanmaja |
| 28 | 1331 | Brassica rapa | 10 | Revonneva-Ruonneva |
| 28 | 1610 | Ribes nigrum | 10 | Revonneva-Ruonneva |
| 29 | 1610 | Dactylis glomerata | 1 | Suomijärvi |
| 29 | 20805 | Festuca trachyphylla | 1 | Suomijärvi |
| 31 | 42203 | Phleum nodosum | 2 | Lohjanjärvi |
| 31 | 24735 | Phleum nodosum | 7 | Lohjanjärvi |
| 32 | 24735 | Fragaria vesca | 12 | Riisitunturi National park |
| 32 | 860 | Rubus arcticus | 4 | Riisitunturi National park |
| 33 | 860 | Lactuca sibirica | 5 | Värriö |
| 33 | 928 | Phalaroides arundinacea | 5 | Värriö |
| 34 | 928 | Mentha arvensis | 14 | Kutujoki |
| 34 | 81833 | Ribes nigrum | 9 | Kutujoki |
| 35 | 81833 | Phalaroides arundinacea | 4 | Issakka |
| 35 | 966 | Trifolium hybridum | 4 | Issakka |
| 36 | 42203 | Schedonorus pratensis | 9 | Liimanninkoski |
| 37 | 43401 | Lactuca sibirica | 6 | Päätyeenlahti |
| 38 | 45483 | Lolium multiflorum | 7 | Siikalahti |
| 39 | 966 | Allium schoenoprasum | 13 | Kielajoki |
| 40 | 83162 | Allium schoenoprasum | 2 | Lövkullaudden |
| 41 | 843 | Allium schoenoprasum | 7 | Isojärvi national park |
| 42 | 83164 | Festuca nigrescens | 13 | Järämä |
| 43 | 889 | Lactuca sibirica | 7 | Lohijoki |
| 44 | 1149 | Barbarea stricta | 6 | Ruosmesuo-Hanhisuo |
| 45 | 849 | Festuca trachyphylla | 13 | Lemmenjoki National Park |
| 46 | 1512 | Brassica rapa | 15 | Kiiminki |
| 47 | 83163 | Poa alpina | 2 | Kalddasjohka |
| 48 | 45761 | Festuca trachyphylla | 7 | Valklammi |
| 49 | 1641 | Carum carvi | 9 | Suuripää - Saaranoja |
| 50 | 862 | Malus sylvestris | 3 | Archipelago Sea National Park |
| 51 | 1962 | Schedonorus pratensis | 5 | Käsivarsi Wilderness Area |
| 52 | 89186 | Trifolium hybridum | 15 | Pirnesoja |
| 53 | 1403 | Festuca trachyphylla | 2 | Päijänne |
| 54 | 93419 | Lactuca sibirica | 4 | Sorsatunturi conservation area |
| 55 | 92822 | Mentha arvensis | 13 | Hossa National Park |

| COMPLEMENTARY SITE | SITE ID | COMPLEMENTARY SPECIES | ELC CATEGORY | SITE NAME |
|---|---|---|---|---|
| 56 | 64798 | Festuca trachyphylla | 9 | Louhensuo conservation area |
| 57 | 25750 | Lactuca sibirica | 10 | Pisavaara nature reserve |
| 58 | 27652 | Mentha arvensis | 1 | Patvinsuo |
| 59 | 1647 | Ribes spicatum | 4 | Särkivaara - Löyhkönen |
| | | | | |

Table S3. Mainland Finland and Åland Islands *in situ* complementary sites and species (Site ID number provided by the World Database of Protected Areas)

| COMPLEMENTARY SITE | SITE ID | COMPLEMENTARY SPECIES | ELC CATEGORY | SITE NAME |
|---|---|---|---|---|
| 1 | 555543215 | Allium schoenoprasum | 7 | Tammisaari and Hanko Archipelago |
| 1 | 555543215 | Allium schoenoprasum | 8 | Tammisaari and Hanko Archipelago |
| 1 | 555543215 | Asparagus officinalis | 7 | Tammisaari and Hanko Archipelago |
| 1 | 555543215 | Barbarea stricta | 7 | Tammisaari and Hanko Archipelago |
| 1 | 555543215 | Barbarea stricta | 8 | Tammisaari and Hanko Archipelago |
| 1 | 555543215 | Barbarea vulgaris | 7 | Tammisaari and Hanko Archipelago |
| 1 | 555543215 | Barbarea vulgaris | 8 | Tammisaari and Hanko Archipelago |
| 1 | 555543215 | Brassica rapa | 7 | Tammisaari and Hanko Archipelago |
| 1 | 555543215 | Bromopsis inermis | 8 | Tammisaari and Hanko Archipelago |
| 1 | 555543215 | Carum carvi | 7 | Tammisaari and Hanko Archipelago |
| 1 | 555543215 | Carum carvi | 8 | Tammisaari and Hanko Archipelago |
| 1 | 555543215 | Corylus avellana | 7 | Tammisaari and Hanko Archipelago |
| 1 | 555543215 | Corylus avellana | 8 | Tammisaari and Hanko Archipelago |
| 1 | 555543215 | Crambe maritima | 8 | Tammisaari and Hanko Archipelago |
| 1 | 555543215 | Dactylis glomerata | 7 | Tammisaari and Hanko Archipelago |
| 1 | 555543215 | Dactylis glomerata | 8 | Tammisaari and Hanko Archipelago |
| 1 | 555543215 | Festuca ovina | 7 | Tammisaari and Hanko Archipelago |
| 1 | 555543215 | Festuca ovina | 8 | Tammisaari and Hanko Archipelago |
| 1 | 555543215 | Festuca rubra | 7 | Tammisaari and Hanko Archipelago |
| 1 | 555543215 | Festuca rubra | 8 | Tammisaari and Hanko Archipelago |
| 1 | 555543215 | Fragaria moschata | 7 | Tammisaari and Hanko Archipelago |
| 1 | 555543215 | Fragaria vesca | 7 | Tammisaari and Hanko Archipelago |
| 1 | 555543215 | Fragaria vesca | 8 | Tammisaari and Hanko Archipelago |
| 1 | 555543215 | Lactuca tatarica | 8 | Tammisaari and Hanko Archipelago |
| 1 | 555543215 | Lolium perenne | 7 | Tammisaari and Hanko Archipelago |
| 1 | 555543215 | Lolium perenne | 8 | Tammisaari and Hanko Archipelago |
| 1 | 555543215 | Malus sylvestris | 8 | Tammisaari and Hanko Archipelago |
| 1 | 555543215 | Medicago lupulina | 7 | Tammisaari and Hanko Archipelago |
| 1 | 555543215 | Mentha arvensis | 7 | Tammisaari and Hanko Archipelago |
| 1 | 555543215 | Mentha arvensis | 8 | Tammisaari and Hanko Archipelago |
| 1 | 555543215 | Phalaroides arundinacea | 7 | Tammisaari and Hanko Archipelago |
| 1 | 555543215 | Phalaroides arundinacea | 8 | Tammisaari and Hanko Archipelago |
| 1 | 555543215 | Phleum pratense | 7 | Tammisaari and Hanko Archipelago |
| 1 | 555543215 | Phleum pratense | 8 | Tammisaari and Hanko Archipelago |
| 1 | 555543215 | Poa pratensis | 7 | Tammisaari and Hanko Archipelago |
| 1 | 555543215 | Poa pratensis | 8 | Tammisaari and Hanko Archipelago |
| 1 | 555543215 | Ribes nigrum | 7 | Tammisaari and Hanko Archipelago |
| 1 | 555543215 | Ribes nigrum | 8 | Tammisaari and Hanko Archipelago |
| 1 | 555543215 | Ribes spicatum | 7 | Tammisaari and Hanko Archipelago |
| 1 | 555543215 | Ribes uva-crispa | 7 | Tammisaari and Hanko Archipelago |

| COMPLEMENTARY SITE | SITE ID | COMPLEMENTARY SPECIES | ELC CATEGORY | SITE NAME |
|---|---|---|---|---|
| 1 | 555543215 | Rubus chamaemorus | 8 | Tammisaari and Hanko Archipelago |
| 1 | 555543215 | Rubus idaeus | 7 | Tammisaari and Hanko Archipelago |
| 1 | 555543215 | Rubus idaeus | 8 | Tammisaari and Hanko Archipelago |
| 1 | 555543215 | Schedonorus pratensis | 7 | Tammisaari and Hanko Archipelago |
| 1 | 555543215 | Schedonorus pratensis | 8 | Tammisaari and Hanko Archipelago |
| 1 | 555543215 | Trifolium hybridum | 7 | Tammisaari and Hanko Archipelago |
| 1 | 555543215 | Trifolium hybridum | 8 | Tammisaari and Hanko Archipelago |
| 1 | 555543215 | Trifolium pratense | 7 | Tammisaari and Hanko Archipelago |
| 1 | 555543215 | Trifolium pratense | 8 | Tammisaari and Hanko Archipelago |
| 1 | 555543215 | Trifolium repens | 7 | Tammisaari and Hanko Archipelago |
| 1 | 555543215 | Trifolium repens | 8 | Tammisaari and Hanko Archipelago |
| 1 | 555543215 | Vaccinium myrtillus | 7 | Tammisaari and Hanko Archipelago |
| 1 | 555543215 | Vaccinium myrtillus | 8 | Tammisaari and Hanko Archipelago |
| 1 | 555543215 | Vaccinium oxycoccos | 7 | Tammisaari and Hanko Archipelago |
| 1 | 555543215 | Vaccinium uliginosum | 8 | Tammisaari and Hanko Archipelago |
| 1 | 555543215 | Vaccinium vitis-idaea | 7 | Tammisaari and Hanko Archipelago |
| 1 | 555543215 | Vaccinium vitis-idaea | 8 | Tammisaari and Hanko Archipelago |
| 2 | 555580012 | Festuca ovina | 10 | Urho Kekkonen National Park |
| 2 | 555580012 | Festuca ovina | 4 | Urho Kekkonen National Park |
| 2 | 555580012 | Festuca ovina | 5 | Urho Kekkonen National Park |
| 2 | 555580012 | Festuca ovina | 9 | Urho Kekkonen National Park |
| 2 | 555580012 | Festuca rubra | 4 | Urho Kekkonen National Park |
| 2 | 555580012 | Festuca rubra | 5 | Urho Kekkonen National Park |
| 2 | 555580012 | Lactuca sibirica | 4 | Urho Kekkonen National Park |
| 2 | 555580012 | Lactuca sibirica | 5 | Urho Kekkonen National Park |
| 2 | 555580012 | Phalaroides arundinacea | 4 | Urho Kekkonen National Park |
| 2 | 555580012 | Phalaroides arundinacea | 5 | Urho Kekkonen National Park |
| 2 | 555580012 | Phalaroides arundinacea | 9 | Urho Kekkonen National Park |
| 2 | 555580012 | Phleum pratense | 5 | Urho Kekkonen National Park |
| 2 | 555580012 | Poa alpina | 10 | Urho Kekkonen National Park |
| 2 | 555580012 | Poa alpina | 5 | Urho Kekkonen National Park |
| 2 | 555580012 | Poa pratensis | 5 | Urho Kekkonen National Park |
| 2 | 555580012 | Ribes nigrum | 5 | Urho Kekkonen National Park |
| 2 | 555580012 | Ribes spicatum | 4 | Urho Kekkonen National Park |
| 2 | 555580012 | Ribes spicatum | 5 | Urho Kekkonen National Park |
| 2 | 555580012 | Ribes spicatum | 9 | Urho Kekkonen National Park |
| 2 | 555580012 | Rubus arcticus | 10 | Urho Kekkonen National Park |
| 2 | 555580012 | Rubus arcticus | 4 | Urho Kekkonen National Park |
| 2 | 555580012 | Rubus arcticus | 5 | Urho Kekkonen National Park |
| 2 | 555580012 | Rubus arcticus | 9 | Urho Kekkonen National Park |
| 2 | 555580012 | Rubus chamaemorus | 10 | Urho Kekkonen National Park |
| 2 | 555580012 | Rubus chamaemorus | 4 | Urho Kekkonen National Park |
| 2 | 555580012 | Rubus chamaemorus | 5 | Urho Kekkonen National Park |
| 2 | 555580012 | Rubus chamaemorus | 9 | Urho Kekkonen National Park |

| COMPLEMENTARY SITE | SITE ID | COMPLEMENTARY SPECIES | ELC CATEGORY | SITE NAME |
|---|---|---|---|---|
| 2 | 555580012 | Rubus idaeus | 4 | Urho Kekkonen National Park |
| 2 | 555580012 | Rubus idaeus | 5 | Urho Kekkonen National Park |
| 2 | 555580012 | Trifolium hybridum | 5 | Urho Kekkonen National Park |
| 2 | 555580012 | Trifolium pratense | 5 | Urho Kekkonen National Park |
| 2 | 555580012 | Trifolium repens | 4 | Urho Kekkonen National Park |
| 2 | 555580012 | Trifolium repens | 5 | Urho Kekkonen National Park |
| 2 | 555580012 | Vaccinium microcarpum | 10 | Urho Kekkonen National Park |
| 2 | 555580012 | Vaccinium microcarpum | 4 | Urho Kekkonen National Park |
| 2 | 555580012 | Vaccinium microcarpum | 5 | Urho Kekkonen National Park |
| 2 | 555580012 | Vaccinium myrtillus | 10 | Urho Kekkonen National Park |
| 2 | 555580012 | Vaccinium myrtillus | 4 | Urho Kekkonen National Park |
| 2 | 555580012 | Vaccinium myrtillus | 5 | Urho Kekkonen National Park |
| 2 | 555580012 | Vaccinium myrtillus | 9 | Urho Kekkonen National Park |
| 2 | 555580012 | Vaccinium oxycoccos | 10 | Urho Kekkonen National Park |
| 2 | 555580012 | Vaccinium oxycoccos | 4 | Urho Kekkonen National Park |
| 2 | 555580012 | Vaccinium oxycoccos | 5 | Urho Kekkonen National Park |
| 2 | 555580012 | Vaccinium uliginosum | 10 | Urho Kekkonen National Park |
| 2 | 555580012 | Vaccinium uliginosum | 4 | Urho Kekkonen National Park |
| 2 | 555580012 | Vaccinium uliginosum | 5 | Urho Kekkonen National Park |
| 2 | 555580012 | Vaccinium uliginosum | 9 | Urho Kekkonen National Park |
| 2 | 555580012 | Vaccinium vitis-idaea | 10 | Urho Kekkonen National Park |
| 2 | 555580012 | Vaccinium vitis-idaea | 4 | Urho Kekkonen National Park |
| 2 | 555580012 | Vaccinium vitis-idaea | 5 | Urho Kekkonen National Park |
| 2 | 555580012 | Vaccinium vitis-idaea | 9 | Urho Kekkonen National Park |
| 3 | 555525183 | Barbarea stricta | 3 | Kiiminki River |
| 3 | 555525183 | Barbarea vulgaris | 1 | Kiiminki River |
| 3 | 555525183 | Barbarea vulgaris | 3 | Kiiminki River |
| 3 | 555525183 | Brassica rapa | 3 | Kiiminki River |
| 3 | 555525183 | Carum carvi | 1 | Kiiminki River |
| 3 | 555525183 | Carum carvi | 3 | Kiiminki River |
| 3 | 555525183 | Festuca ovina | 1 | Kiiminki River |
| 3 | 555525183 | Festuca ovina | 3 | Kiiminki River |
| 3 | 555525183 | Festuca rubra | 1 | Kiiminki River |
| 3 | 555525183 | Festuca rubra | 3 | Kiiminki River |
| 3 | 555525183 | Fragaria vesca | 1 | Kiiminki River |
| 3 | 555525183 | Fragaria vesca | 3 | Kiiminki River |
| 3 | 555525183 | Lactuca sibirica | 1 | Kiiminki River |
| 3 | 555525183 | Mentha arvensis | 1 | Kiiminki River |
| 3 | 555525183 | Mentha arvensis | 3 | Kiiminki River |
| 3 | 555525183 | Phalaroides arundinacea | 1 | Kiiminki River |
| 3 | 555525183 | Phalaroides arundinacea | 3 | Kiiminki River |
| 3 | 555525183 | Phleum pratense | 1 | Kiiminki River |
| 3 | 555525183 | Phleum pratense | 3 | Kiiminki River |
| 3 | 555525183 | Poa pratensis | 1 | Kiiminki River |

| COMPLEMENTARY SITE | SITE ID | COMPLEMENTARY SPECIES | ELC CATEGORY | SITE NAME |
|---|---|---|---|---|
| 3 | 555525183 | Poa pratensis | 3 | Kiiminki River |
| 3 | 555525183 | Ribes nigrum | 1 | Kiiminki River |
| 3 | 555525183 | Ribes spicatum | 3 | Kiiminki River |
| 3 | 555525183 | Rubus arcticus | 1 | Kiiminki River |
| 3 | 555525183 | Rubus arcticus | 3 | Kiiminki River |
| 3 | 555525183 | Rubus chamaemorus | 1 | Kiiminki River |
| 3 | 555525183 | Rubus chamaemorus | 3 | Kiiminki River |
| 3 | 555525183 | Rubus idaeus | 1 | Kiiminki River |
| 3 | 555525183 | Rubus idaeus | 3 | Kiiminki River |
| 3 | 555525183 | Schedonorus pratensis | 3 | Kiiminki River |
| 3 | 555525183 | Trifolium pratense | 1 | Kiiminki River |
| 3 | 555525183 | Trifolium pratense | 3 | Kiiminki River |
| 3 | 555525183 | Trifolium repens | 1 | Kiiminki River |
| 3 | 555525183 | Trifolium repens | 3 | Kiiminki River |
| 3 | 555525183 | Vaccinium myrtillus | 1 | Kiiminki River |
| 3 | 555525183 | Vaccinium myrtillus | 3 | Kiiminki River |
| 3 | 555525183 | Vaccinium oxycoccos | 1 | Kiiminki River |
| 3 | 555525183 | Vaccinium oxycoccos | 3 | Kiiminki River |
| 3 | 555525183 | Vaccinium uliginosum | 1 | Kiiminki River |
| 3 | 555525183 | Vaccinium uliginosum | 3 | Kiiminki River |
| 3 | 555525183 | Vaccinium vitis-idaea | 1 | Kiiminki River |
| 3 | 555525183 | Vaccinium vitis-idaea | 3 | Kiiminki River |
| 4 | 555524483 | Barbarea stricta | 6 | Evo |
| 4 | 555524483 | Dactylis glomerata | 6 | Evo |
| 4 | 555524483 | Festuca ovina | 6 | Evo |
| 4 | 555524483 | Schedonorus pratensis | 6 | Evo |
| 4 | 555524483 | Festuca rubra | 6 | Evo |
| 4 | 555524483 | Fragaria vesca | 6 | Evo |
| 4 | 555524483 | Phalaroides arundinacea | 6 | Evo |
| 4 | 555524483 | Phleum pratense | 6 | Evo |
| 4 | 555524483 | Poa pratensis | 6 | Evo |
| 4 | 555524483 | Ribes nigrum | 6 | Evo |
| 4 | 555524483 | Ribes spicatum | 8 | Evo |
| 4 | 555524483 | Ribes uva-crispa | 6 | Evo |
| 4 | 555524483 | Ribes uva-crispa | 8 | Evo |
| 4 | 555524483 | Rubus chamaemorus | 6 | Evo |
| 4 | 555524483 | Rubus idaeus | 6 | Evo |
| 4 | 555524483 | Trifolium pratense | 6 | Evo |
| 4 | 555524483 | Trifolium repens | 6 | Evo |
| 4 | 555524483 | Vaccinium myrtillus | 6 | Evo |
| 4 | 555524483 | Vaccinium oxycoccos | 6 | Evo |
| 4 | 555524483 | Vaccinium oxycoccos | 8 | Evo |
| 4 | 555524483 | Vaccinium uliginosum | 6 | Evo |
| 4 | 555524483 | Vaccinium vitis-idaea | 6 | Evo |

| COMPLEMENTARY SITE | SITE ID | COMPLEMENTARY SPECIES | ELC CATEGORY | SITE NAME |
|---|---|---|---|---|
| 5 | 555524483 | Barbarea stricta | 5 | Oulanka National Park |
| 5 | 902780 | Dactylis glomerata | 5 | Oulanka National Park |
| 5 | 902780 | Festuca rubra | 10 | Oulanka National Park |
| 5 | 902780 | Festuca trachyphylla | 5 | Oulanka National Park |
| 5 | 902780 | Fragaria vesca | 5 | Oulanka National Park |
| 5 | 902780 | Lolium perenne | 5 | Oulanka National Park |
| 5 | 902780 | Poa pratensis | 10 | Oulanka National Park |
| 5 | 902780 | Rubus idaeus | 10 | Oulanka National Park |
| 5 | 902780 | Schedonorus pratensis | 5 | Oulanka National Park |
| 5 | 902780 | Trifolium pratense | 10 | Oulanka National Park |
| 5 | 902780 | Trifolium repens | 10 | Oulanka National Park |
| 6 | 555524301 | Festuca trachyphylla | 7 | Hyyppärä |
| 6 | 555524301 | Festuca trachyphylla | 8 | Hyyppärä |
| 6 | 555524301 | Fragaria moschata | 8 | Hyyppärä |
| 6 | 555524301 | Rubus arcticus | 7 | Hyyppärä |
| 6 | 555524301 | Rubus arcticus | 8 | Hyyppärä |
| 6 | 555524301 | Rubus chamaemorus | 7 | Hyyppärä |
| 6 | 555524301 | Vaccinium microcarpum | 7 | Hyyppärä |
| 6 | 555524301 | Vaccinium microcarpum | 8 | Hyyppärä |
| 6 | 555524301 | Vaccinium uliginosum | 7 | Hyyppärä |
| 7 | 555543221 | Festuca rubra | 2 | Rahja Archipelago |
| 7 | 555543221 | Fragaria vesca | 2 | Rahja Archipelago |
| 7 | 555543221 | Phalaroides arundinacea | 2 | Rahja Archipelago |
| 7 | 555543221 | Rubus arcticus | 2 | Rahja Archipelago |
| 7 | 555543221 | Rubus chamaemorus | 2 | Rahja Archipelago |
| 7 | 555543221 | Vaccinium myrtillus | 2 | Rahja Archipelago |
| 7 | 555543221 | Vaccinium vitis-idaea | 2 | Rahja Archipelago |
| 8 | 555525477 | Barbarea stricta | 4 | Ounas River |
| 8 | 555525477 | Brassica rapa | 4 | Ounas River |
| 8 | 555525477 | Festuca trachyphylla | 4 | Ounas River |
| 8 | 555525477 | Phleum pratense | 4 | Ounas River |
| 8 | 555525477 | Poa pratensis | 4 | Ounas River |
| 8 | 555525477 | Trifolium pratense | 4 | Ounas River |
| 9 | 902791 | Barbarea vulgaris | 6 | Torronsuo National Park |
| 9 | 902791 | Brassica rapa | 6 | Torronsuo National Park |
| 9 | 902791 | Carum carvi | 6 | Torronsuo National Park |
| 9 | 902791 | Rubus arcticus | 6 | Torronsuo National Park |
| 9 | 902791 | Trifolium hybridum | 6 | Torronsuo National Park |
| 9 | 902791 | Vaccinium microcarpum | 6 | Torronsuo National Park |
| 10 | 555580240 | Festuca rubra | 9 | South Kuusamo forests |
| 10 | 555580240 | Trifolium pratense | 9 | South Kuusamo forests |
| 10 | 555580240 | Trifolium repens | 9 | South Kuusamo forests |
| 10 | 555580240 | Vaccinium microcarpum | 9 | South Kuusamo forests |
| 10 | 555580240 | Vaccinium oxycoccos | 9 | South Kuusamo forests |

| COMPLEMENTARY SITE | SITE ID | COMPLEMENTARY SPECIES | ELC CATEGORY | SITE NAME |
|---|---|---|---|---|
| 11 | 555524496 | Armoracia rusticana | 8 | Kukkiajärvi |
| 11 | 555524496 | Brassica rapa | 8 | Kukkiajärvi |
| 11 | 555524496 | Lolium multiflorum | 8 | Kukkiajärvi |
| 11 | 555524496 | Medicago sativa | 8 | Kukkiajärvi |
| 12 | 555580002 | Fragaria vesca | 10 | Värriö |
| 12 | 555580002 | Lactuca sibirica | 10 | Värriö |
| 12 | 555580002 | Phalaroides arundinacea | 10 | Värriö |
| 12 | 555580002 | Ribes spicatum | 10 | Värriö |
| 13 | 555525566 | Armoracia rusticana | 7 | Västra Espholm |
| 13 | 555525566 | Medicago sativa | 7 | Västra Espholm |
| 13 | 555525566 | Rubus caesius | 7 | Västra Espholm |
| 13 | 555525566 | Sinapis arvensis | 7 | Västra Espholm |
| 14 | 149649 | Barbarea stricta | 10 | Saana conservation area |
| 14 | 149649 | Festuca nigrescens | 10 | Saana conservation area |
| 14 | 149649 | Lolium perenne | 10 | Saana conservation area |
| 15 | 902757 | Lactuca sibirica | 6 | Rääkkylä ja Kitee lakes |
| 15 | 902757 | Mentha arvensis | 6 | Rääkkylä ja Kitee lakes |
| 16 | 555579976 | Barbarea vulgaris | 5 | Pallas-Ounastunturi |
| 16 | 555579976 | Carum carvi | 5 | Pallas-Ounastunturi |
| 16 | 555579976 | Poa alpina | 4 | Pallas-Ounastunturi |
| 17 | 555543214 | Crambe maritima | 7 | Archipelago Sea |
| 17 | 555543214 | Fragaria viridis | 7 | Archipelago Sea |
| 17 | 555543214 | Malus sylvestris | 7 | Archipelago Sea |
| 18 | 555525221 | Ribes nigrum | 3 | Pitkäsneva |
| 18 | 555525221 | Vaccinium microcarpum | 1 | Pitkäsneva |
| 18 | 555525221 | Vaccinium microcarpum | 3 | Pitkäsneva |
| 19 | 555525292 | Festuca trachyphylla | 9 | Kinnussuo - Mustinsuo |
| 19 | 555525292 | Phleum pratense | 9 | Kinnussuo - Mustinsuo |
| 19 | 555525292 | Poa pratensis | 9 | Kinnussuo - Mustinsuo |
| 20 | 555524242 | Asparagus officinalis | 8 | Lohjanjärvi area |
| 20 | 555524242 | Phleum nodosum | 8 | Lohjanjärvi area |
| 21 | 555525143 | Dactylis glomerata | 3 | Lestijoki |
| 21 | 555525143 | Trifolium hybridum | 3 | Lestijoki |
| 22 | 555579982 | Carum carvi | 2 | Perämeri islands |
| 22 | 555579982 | Phleum pratense | 2 | Perämeri islands |
| 23 | 555579975 | Schedonorus pratensis | 10 | Käsivarsi Wildeness area |
| 23 | 555579975 | Trifolium hybridum | 10 | Käsivarsi Wildeness area |
| 24 | 555525199 | Fragaria vesca | 4 | Paljakka |
| 24 | 555525199 | Ribes nigrum | 4 | Paljakka |
| 25 | 555524541 | Lactuca sibirica | 8 | Liesjärvi |
| 26 | 555525515 | Allium schoenoprasum | 5 | Paistunturi Wilderness area |
| 27 | 555580055 | Bromopsis inermis | 7 | Ruissalo |
| 28 | 555580018 | Allium schoenoprasum | 10 | Kaldoaivi Wilderness area |
| 29 | 388514 | Lolium multiflorum | 7 | Kalkkikallio conservation area |

| COMPLEMENTARY SITE | SITE ID | COMPLEMENTARY SPECIES | ELC CATEGORY | SITE NAME |
|---|---|---|---|---|
| 30 | 555524330 | Medicago lupulina | 8 | Pomponrahka |
| 31 | 555524355 | Phleum nodosum | 7 | Rekijoki valley |
| 32 | 555525358 | Mentha arvensis | 5 | Vasonniemi ja Pahalammenpuro |
| 33 | 151068 | Lactuca sibirica | 3 | Tornio bird conservation area |
| 34 | 555525516 | Ribes nigrum | 10 | Pulmankijärvi |
| 35 | 555563330 | Ribes spicatum | 6 | Lehtola |
| 36 | 555580272 | Brassica rapa | 1 | Revonneva-Ruonneva |
| 37 | 555525258 | Ribes spicatum | 1 | Kurimonkoski meadows |
| 38 | 329930 | Corylus avellana | 6 | Myllyharju conservation area |
| 39 | 555538774 | Festuca trachyphylla | 6 | Suomijärvi |
| 40 | 555589893 | Lolium perenne | 3 | Riutta seaside meadow |
| 41 | 555525208 | Schedonorus pratensis | 3 | Muhos- ja Poikajoki |
| 42 | 555580235 | Phleum pratense | 10 | Valtavaara - Pyhävaara |
| 43 | 555525507 | Trifolium hybridum | 1 | Vaarajänkkä-Rovajänkkä |
| 44 | 555525373 | Schedonorus pratensis | 4 | Jylkkyvaara ja Jylkynsuo |
| 45 | 555580285 | Rubus idaeus | 9 | Juortanansalo area |
| 46 | 555538773 | Sinapis arvensis | 8 | Viurilanlahti |
| 47 | 555525576 | Mentha aquatica | 7 | Gloviken |
| 48 | 555525530 | Prunus spinosa | 7 | Nåtö - Jungfruskär |

Table S4. *Ex situ* gap analysis - complementary species/ELC in collecting sites. (Site ID number provided by the World Database of Protected Areas)

| COMPLEMENTARY COLLECTING SITE NUMBER | SITE ID | COMPLEMENTARY SPECIES | ELC CATEGORY |
|---|---|---|---|
| 1 | 9040 | Allium schoenoprasum | 12 |
| 1 | 9040 | Armoracia rusticana | 3 |
| 1 | 9040 | Barbarea stricta | 3 |
| 1 | 9040 | Barbarea vulgaris | 3 |
| 1 | 9040 | Brassica rapa | 2 |
| 1 | 9040 | Brassica rapa | 3 |
| 1 | 9040 | Carum carvi | 10 |
| 1 | 9040 | Corylus avellana | 3 |
| 1 | 9040 | Dactylis glomerata | 11 |
| 1 | 9040 | Dactylis glomerata | 12 |
| 1 | 9040 | Dactylis glomerata | 13 |
| 1 | 9040 | Fragaria moschata | 3 |
| 1 | 9040 | Fragaria vesca | 3 |
| 1 | 9040 | Lolium perenne | 3 |
| 1 | 9040 | Mentha arvensis | 3 |
| 1 | 9040 | Ribes nigrum | 10 |
| 1 | 9040 | Ribes nigrum | 15 |
| 1 | 9040 | Ribes spicatum | 4 |
| 1 | 9040 | Ribes spicatum | 5 |
| 1 | 9040 | Ribes uva-crispa | 3 |
| 1 | 9040 | Rubus arcticus | 3 |
| 1 | 9040 | Rubus chamaemorus | 3 |
| 1 | 9040 | Rubus idaeus | 3 |
| 1 | 9040 | Trifolium hybridum | 5 |
| 1 | 9040 | Vaccinium myrtillus | 3 |
| 1 | 9040 | Vaccinium myrtillus | 5 |
| 1 | 9040 | Vaccinium uliginosum | 3 |
| 1 | 9040 | Vaccinium vitis-idaea | 1 |
| 1 | 9040 | Vaccinium vitis-idaea | 2 |
| 2 | 16637 | Armoracia rusticana | 8 |
| 2 | 16637 | Barbarea stricta | 7 |
| 2 | 16637 | Barbarea stricta | 8 |
| 2 | 16637 | Barbarea vulgaris | 8 |
| 2 | 16637 | Bromopsis inermis | 7 |
| 2 | 16637 | Bromopsis inermis | 8 |
| 2 | 16637 | Carum carvi | 4 |
| 2 | 16637 | Chenopodium ficifolium | 8 |
| 2 | 16637 | Erucastrum gallicum | 7 |

| COMPLEMENTARY COLLECTING SITE NUMBER | SITE ID | COMPLEMENTARY SPECIES | ELC CATEGORY |
|---|---|---|---|
| 2 | 16637 | Erucastrum gallicum | 8 |
| 2 | 16637 | Festuca ovina | 5 |
| 2 | 16637 | Festuca trachyphylla | 7 |
| 2 | 16637 | Festuca trachyphylla | 8 |
| 2 | 16637 | Lactuca sibirica | 7 |
| 2 | 16637 | Mentha arvensis | 8 |
| 2 | 16637 | Ribes nigrum | 2 |
| 2 | 16637 | Ribes nigrum | 7 |
| 2 | 16637 | Ribes nigrum | 8 |
| 2 | 16637 | Ribes spicatum | 3 |
| 2 | 16637 | Vaccinium microcarpum | 7 |
| 2 | 16637 | Vaccinium microcarpum | 8 |
| 2 | 16637 | Vaccinium oxycoccos | 2 |
| 2 | 16637 | Vaccinium vitis-idaea | 8 |
| 3 | 8498 | Carum carvi | 3 |
| 3 | 8498 | Dactylis glomerata | 5 |
| 3 | 8498 | Ribes nigrum | 13 |
| 3 | 8498 | Ribes nigrum | 5 |
| 3 | 8498 | Ribes spicatum | 9 |
| 3 | 8498 | Rubus chamaemorus | 10 |
| 3 | 8498 | Rubus chamaemorus | 5 |
| 3 | 8498 | Schedonorus pratensis | 1 |
| 3 | 8498 | Schedonorus pratensis | 2 |
| 3 | 8498 | Trifolium repens | 6 |
| 3 | 8498 | Vaccinium myrtillus | 6 |
| 3 | 8498 | Vaccinium myrtillus | 7 |
| 3 | 8498 | Vaccinium oxycoccos | 4 |
| 3 | 8498 | Vaccinium oxycoccos | 5 |
| 3 | 8498 | Vaccinium vitis-idaea | 10 |
| 3 | 8498 | Vaccinium vitis-idaea | 9 |
| 4 | 17560 | Armoracia rusticana | 7 |
| 4 | 17560 | Barbarea vulgaris | 7 |
| 4 | 17560 | Carum carvi | 5 |
| 4 | 17560 | Corylus avellana | 7 |
| 4 | 17560 | Dactylis glomerata | 1 |
| 4 | 17560 | Dactylis glomerata | 3 |
| 4 | 17560 | Festuca ovina | 4 |
| 4 | 17560 | Festuca rubra | 1 |
| 4 | 17560 | Fragaria vesca | 7 |
| 4 | 17560 | Mentha arvensis | 7 |
| 4 | 17560 | Rubus arcticus | 7 |
| 4 | 17560 | Rubus idaeus | 7 |
| 4 | 17560 | Schedonorus pratensis | 5 |

| COMPLEMENTARY COLLECTING SITE NUMBER | SITE ID | COMPLEMENTARY SPECIES | ELC CATEGORY |
|---|---|---|---|
| 4 | 17560 | Vaccinium myrtillus | 10 |
| 4 | 17560 | Vaccinium oxycoccos | 3 |
| 4 | 17560 | Vaccinium vitis-idaea | 6 |
| 5 | 9791 | Barbarea stricta | 1 |
| 5 | 9791 | Bromopsis inermis | 3 |
| 5 | 9791 | Festuca trachyphylla | 1 |
| 5 | 9791 | Festuca trachyphylla | 3 |
| 5 | 9791 | Mentha arvensis | 1 |
| 5 | 9791 | Ribes nigrum | 14 |
| 5 | 9791 | Rubus arcticus | 1 |
| 5 | 9791 | Rubus chamaemorus | 1 |
| 5 | 9791 | Rubus idaeus | 1 |
| 5 | 9791 | Schedonorus pratensis | 12 |
| 5 | 9791 | Trifolium repens | 1 |
| 5 | 9791 | Vaccinium microcarpum | 1 |
| 5 | 9791 | Vaccinium myrtillus | 4 |
| 5 | 9791 | Vaccinium oxycoccos | 7 |
| 5 | 9791 | Vaccinium uliginosum | 1 |
| 5 | 9791 | Vaccinium vitis-idaea | 3 |
| 6 | 14100 | Barbarea vulgaris | 6 |
| 6 | 14100 | Bromopsis inermis | 6 |
| 6 | 14100 | Carum carvi | 6 |
| 6 | 14100 | Fragaria vesca | 6 |
| 6 | 14100 | Fragaria vesca | 8 |
| 6 | 14100 | Poa pratensis | 4 |
| 6 | 14100 | Ribes nigrum | 1 |
| 6 | 14100 | Ribes nigrum | 6 |
| 6 | 14100 | Ribes spicatum | 10 |
| 6 | 14100 | Rubus chamaemorus | 6 |
| 6 | 14100 | Trifolium hybridum | 8 |
| 6 | 14100 | Trifolium hybridum | 9 |
| 6 | 14100 | Vaccinium myrtillus | 9 |
| 7 | 5235 | Barbarea stricta | 5 |
| 7 | 5235 | Carum carvi | 7 |
| 7 | 5235 | Cichorium intybus | 5 |
| 7 | 5235 | Corylus avellana | 4 |
| 7 | 5235 | Corylus avellana | 5 |
| 7 | 5235 | Ribes spicatum | 2 |
| 7 | 5235 | Rubus arcticus | 4 |
| 7 | 5235 | Rubus arcticus | 5 |
| 7 | 5235 | Vaccinium myrtillus | 1 |
| 7 | 5235 | Vaccinium oxycoccos | 10 |
| 7 | 5235 | Vaccinium oxycoccos | 9 |

| COMPLEMENTARY COLLECTING SITE NUMBER | SITE ID | COMPLEMENTARY SPECIES | ELC CATEGORY |
|---|---|---|---|
| 7 | 5235 | Vaccinium vitis-idaea | 4 |
| 7 | 5235 | Vaccinium vitis-idaea | 5 |
| 8 | 12594 | Brassica rapa | 1 |
| 8 | 7854 | Festuca ovina | 10 |
| 8 | 7854 | Festuca rubra | 6 |
| 8 | 7854 | Lolium perenne | 2 |
| 8 | 7854 | Mentha arvensis | 2 |
| 8 | 7854 | Phleum pratense | 8 |
| 8 | 7854 | Ribes nigrum | 16 |
| 8 | 7854 | Rubus idaeus | 2 |
| 8 | 7854 | Schedonorus pratensis | 11 |
| 8 | 7854 | Trifolium hybridum | 10 |
| 8 | 7854 | Vaccinium oxycoccos | 6 |
| 8 | 7854 | Vaccinium oxycoccos | 8 |
| 9 | 9233 | Barbarea vulgaris | 4 |
| 9 | 9233 | Dactylis glomerata | 4 |
| 9 | 9233 | Fragaria vesca | 4 |
| 9 | 9233 | Phleum pratense | 5 |
| 9 | 9233 | Ribes nigrum | 12 |
| 9 | 9233 | Rubus chamaemorus | 4 |
| 9 | 9233 | Rubus chamaemorus | 9 |
| 9 | 9233 | Trifolium hybridum | 6 |
| 9 | 9233 | Vaccinium microcarpum | 9 |
| 10 | 5533 | Fragaria vesca | 5 |
| 10 | 5533 | Lolium perenne | 5 |
| 10 | 5533 | Phalaroides arundinacea | 6 |
| 10 | 5533 | Ribes uva-crispa | 4 |
| 10 | 5533 | Ribes uva-crispa | 5 |
| 10 | 5533 | Rubus idaeus | 5 |
| 10 | 5533 | Vaccinium myrtillus | 2 |
| 10 | 5533 | Vaccinium uliginosum | 4 |
| 10 | 5533 | Vaccinium uliginosum | 5 |
| 11 | 7763 | Barbarea stricta | 10 |
| 11 | 7763 | Carum carvi | 1 |
| 11 | 7763 | Dactylis glomerata | 15 |
| 11 | 7763 | Fragaria vesca | 10 |
| 11 | 7763 | Lolium perenne | 10 |
| 11 | 7763 | Ribes uva-crispa | 10 |
| 11 | 7763 | Trifolium hybridum | 7 |
| 11 | 7763 | Vaccinium uliginosum | 10 |
| 12 | 16856 | Barbarea stricta | 6 |
| 12 | 16856 | Corylus avellana | 6 |
| 12 | 16856 | Corylus avellana | 8 |

| COMPLEMENTARY COLLECTING SITE NUMBER | SITE ID | COMPLEMENTARY SPECIES | ELC CATEGORY |
|---|---|---|---|
| 12 | 16856 | Festuca trachyphylla | 6 |
| 12 | 16856 | Lolium perenne | 6 |
| 12 | 16856 | Lolium perenne | 8 |
| 12 | 16856 | Rubus chamaemorus | 8 |
| 12 | 16856 | Rubus idaeus | 8 |
| 13 | 1929 | Festuca ovina | 1 |
| 13 | 1929 | Festuca rubra | 4 |
| 13 | 1929 | Ribes spicatum | 7 |
| 13 | 1929 | Rubus arcticus | 10 |
| 13 | 1929 | Trifolium pratense | 12 |
| 13 | 1929 | Trifolium repens | 12 |
| 14 | 10085 | Barbarea vulgaris | 1 |
| 14 | 10085 | Bromopsis inermis | 1 |
| 14 | 10085 | Chenopodium ficifolium | 1 |
| 14 | 10085 | Festuca nigrescens | 3 |
| 14 | 10085 | Ribes nigrum | 9 |
| 14 | 10085 | Ribes uva-crispa | 1 |
| 15 | 15495 | Allium schoenoprasum | 2 |
| 15 | 15495 | Asparagus officinalis | 8 |
| 15 | 15495 | Fragaria moschata | 8 |
| 15 | 15495 | Ribes uva-crispa | 8 |
| 15 | 15495 | Vaccinium uliginosum | 8 |
| 16 | 12528 | Lactuca sibirica | 6 |
| 16 | 12528 | Rubus arcticus | 6 |
| 16 | 12528 | Rubus arcticus | 8 |
| 16 | 12528 | Schedonorus pratensis | 7 |
| 16 | 12528 | Vaccinium microcarpum | 6 |
| 17 | 8499 | Barbarea stricta | 9 |
| 17 | 8499 | Barbarea vulgaris | 9 |
| 17 | 8499 | Ribes spicatum | 8 |
| 17 | 8499 | Rubus arcticus | 9 |
| 17 | 8499 | Rubus idaeus | 9 |
| 18 | 7027 | Barbarea vulgaris | 10 |
| 18 | 7027 | Brassica rapa | 5 |
| 18 | 7027 | Mentha arvensis | 10 |
| 18 | 7027 | Rubus idaeus | 10 |
| 19 | 5348 | Barbarea stricta | 4 |
| 19 | 5348 | Brassica rapa | 6 |
| 19 | 5348 | Carum carvi | 8 |
| 19 | 5348 | Ribes spicatum | 1 |
| 20 | 17092 | Rubus chamaemorus | 7 |
| 20 | 17092 | Asparagus officinalis | 7 |
| 20 | 17092 | Crambe maritima | 7 |

| COMPLEMENTARY COLLECTING SITE NUMBER | SITE ID | COMPLEMENTARY SPECIES | ELC CATEGORY |
|---|---|---|---|
| 20 | 17092 | Fragaria moschata | 7 |
| 21 | 7712 | Allium schoenoprasum | 17 |
| 21 | 7712 | Allium schoenoprasum | 14 |
| 21 | 7712 | Allium schoenoprasum | 11 |
| 22 | 4644 | Brassica rapa | 4 |
| 22 | 4644 | Lolium perenne | 4 |
| 22 | 4644 | Rubus idaeus | 4 |
| 23 | 11071 | Barbarea stricta | 2 |
| 23 | 11071 | Ribes nigrum | 11 |
| 23 | 11071 | Rubus arcticus | 2 |
| 24 | 548 | Lactuca sibirica | 10 |
| 24 | 548 | Poa pratensis | 2 |
| 24 | 548 | Ribes nigrum | 4 |
| 25 | 17113 | Cichorium intybus | 8 |
| 25 | 17113 | Lolium multiflorum | 8 |
| 25 | 17113 | Sinapis arvensis | 8 |
| 26 | 13711 | Medicago sativa | 6 |
| 26 | 13711 | Mentha arvensis | 6 |
| 26 | 13711 | Vaccinium uliginosum | 6 |
| 27 | 8044 | Fragaria moschata | 5 |
| 27 | 8044 | Vaccinium microcarpum | 4 |
| 27 | 8044 | Vaccinium microcarpum | 5 |
| 28 | 9937 | Chenopodium ficifolium | 3 |
| 28 | 9937 | Lactuca sibirica | 3 |
| 28 | 9937 | Vaccinium microcarpum | 3 |
| 29 | 10083 | Erucastrum gallicum | 1 |
| 29 | 10083 | Fragaria vesca | 1 |
| 29 | 10083 | Lactuca sibirica | 1 |
| 30 | 15777 | Lactuca sibirica | 8 |
| 30 | 15777 | Vaccinium uliginosum | 7 |
| 31 | 14755 | Medicago lupulina | 8 |
| 31 | 14755 | Mentha aquatica | 8 |
| 32 | 16937 | Allium schoenoprasum | 3 |
| 32 | 16937 | Allium schoenoprasum | 9 |
| 33 | 6814 | Barbarea vulgaris | 5 |
| 33 | 6814 | Bromopsis inermis | 5 |
| 34 | 12473 | Brassica rapa | 11 |
| 34 | 12473 | Festuca nigrescens | 8 |
| 35 | 15367 | Fragaria moschata | 6 |
| 35 | 15367 | Rubus idaeus | 6 |
| 36 | 17352 | Lolium perenne | 7 |
| 36 | 17352 | Medicago lupulina | 7 |
| 37 | 11002 | Fragaria moschata | 9 |

| COMPLEMENTARY COLLECTING SITE NUMBER | SITE ID | COMPLEMENTARY SPECIES | ELC CATEGORY |
|---|---|---|---|
| 37 | 11002 | Fragaria vesca | 9 |
| 38 | 1928 | Trifolium repens | 10 |
| 38 | 1928 | Trifolium hybridum | 1 |
| 39 | 6827 | Mentha arvensis | 4 |
| 39 | 6827 | Mentha arvensis | 5 |
| 40 | 11068 | Corylus avellana | 2 |
| 40 | 11068 | Fragaria vesca | 2 |
| 41 | 8330 | Rubus caesius | 1 |
| 41 | 8330 | Rubus caesius | 3 |
| 42 | 11947 | Diplotaxis muralis | 8 |
| 42 | 11947 | Prunus spinosa | 8 |
| 43 | 10065 | Cichorium intybus | 1 |
| 43 | 10065 | Lolium perenne | 1 |
| 44 | 7123 | Poa alpina | 1 |
| 44 | 7123 | Poa alpina | 3 |
| 45 | 8042 | Festuca trachyphylla | 5 |
| 45 | 8042 | Festuca trachyphylla | 4 |
| 46 | 13355 | Medicago sativa | 8 |
| 47 | 16222 | Medicago lupulina | 6 |
| 48 | 12594 | Allium schoenoprasum | 5 |
| 49 | 8892 | Medicago lupulina | 3 |
| 50 | 3447 | Ribes spicatum | 6 |
| 51 | 7572 | Allium schoenoprasum | 18 |
| 52 | 9767 | Prunus spinosa | 3 |
| 53 | 8598 | Medicago sativa | 3 |
| 54 | 10066 | Cichorium intybus | 3 |
| 55 | 7706 | Mentha aquatica | 3 |
| 56 | 9345 | Corylus avellana | 1 |
| 57 | 6115 | Bromopsis inermis | 4 |
| 58 | 4796 | Cichorium intybus | 4 |
| 59 | 12318 | Brassica rapa | 10 |
| 60 | 9371 | Brassica rapa | 12 |
| 61 | 8935 | Vaccinium microcarpum | 10 |
| 62 | 8209 | Vaccinium uliginosum | 9 |
| 63 | 9515 | Fragaria moschata | 4 |
| 64 | 8196 | Poa alpina | 4 |
| 65 | 17104 | Crambe maritima | 8 |
| 66 | 16489 | Chenopodium ficifolium | 7 |
| 67 | 15050 | Poa alpina | 8 |
| 68 | 13695 | Ribes uva-crispa | 7 |
| 69 | 16564 | Ribes uva-crispa | 6 |
| 70 | 15051 | Fragaria viridis | 8 |
| 71 | 9814 | Fragaria moschata | 1 |

| COMPLEMENTARY COLLECTING SITE NUMBER | SITE ID | COMPLEMENTARY SPECIES | ELC CATEGORY |
|---|---|---|---|
| 72 | 17647 | Medicago sativa | 7 |
| 73 | 16340 | Diplotaxis tenuifolia | 7 |
| 74 | 11007 | Bromopsis inermis | 10 |
| 75 | 16799 | Poa alpina | 7 |
| 76 | 17550 | Armoracia rusticana | 6 |
| 77 | 11651 | Prunus spinosa | 7 |
| 78 | 11944 | Diplotaxis muralis | 7 |
| 79 | 398 | Poa pratensis | 1 |
| 80 | 2462 | Trifolium hybridum | 2 |
| 81 | 2022 | Festuca ovina | 2 |
| 82 | 17211 | Lactuca tatarica | 7 |
| 83 | 17080 | Lolium multiflorum | 7 |
| 84 | 13408 | Poa alpina | 6 |
| 85 | 11067 | Fragaria moschata | 2 |
| 86 | 11211 | Ribes uva-crispa | 2 |
| 87 | 2021 | Festuca rubra | 5 |
| 88 | 11802 | Prunus spinosa | 6 |
| 89 | 11946 | Diplotaxis muralis | 6 |
| 90 | 14895 | Vicia lathyroides | 8 |
| 91 | 16115 | Rubus caesius | 8 |
| 92 | 12719 | Lolium multiflorum | 6 |
| 93 | 12522 | Rubus caesius | 7 |
| 94 | 10968 | Lolium multiflorum | 3 |
| 95 | 8053 | Poa alpina | 10 |
| 96 | 9973 | Fragaria moschata | 10 |
| 97 | 8322 | Asparagus officinalis | 1 |
| 98 | 12375 | Festuca nigrescens | 7 |
| 99 | 3037 | Medicago sativa | 5 |
| 100 | 7175 | Ribes uva-crispa | 9 |
| 101 | 4677 | Trifolium repens | 11 |
| 102 | 7891 | Rubus caesius | 5 |
| 103 | 4680 | Lolium perenne | 9 |
| 104 | 7748 | Poa alpina | 5 |
| 105 | 5236 | Chenopodium ficifolium | 5 |
| 106 | 10381 | Erucastrum gallicum | 3 |
| 107 | 11961 | Cichorium intybus | 6 |
